# Impact of plastic-related chemicals on emotional and behavioral health in children from Poland

**DOI:** 10.1186/s12940-025-01210-6

**Published:** 2025-10-14

**Authors:** Kinga Polańska, Agnieszka Jankowska, Daniel Bury, Rebecca K. Moos, Claudia Pälmke, Joanna Jerzyńska, Joanna Jurewicz, Stephan Bose-O’Reilly, Holger M. Koch, Mercè Garí

**Affiliations:** 1 Department of Environmental and Occupational Health Hazards, Nofer Institute of Occupational Medicine (NIOM), Lodz, Poland; 2 Institute for Prevention and Occupational Medicine of the German Social Accident Insurance - Institute of the Ruhr University Bochum (IPA), Bochum, Germany; 3 Department of Paediatrics and Allergy, Korczak Center, Copernicus Memorial Hospital, Medical University of Lodz (MUL), Lodz, Poland; 4 Department of Toxicology, Medical University of Lodz (MUL), Lodz, Poland; 5 Institute and Clinic for Occupational, Social and Environmental Medicine, University Hospital, LMU Munich, Munich, Germany; 6 Institute of Environmental Assessment and Water Research (IDAEA-CSIC), Barcelona, Catalonia Spain

**Keywords:** Birth cohort, Children’s behavior, Phthalates, Non-phthalate plasticizers, DINCH, DEHTP, Bisphenols, Bayesian mixture modelling, Sex-specific accounting effects

## Abstract

**Background:**

Exposure to phthalates and non-phthalate plasticizers as well as bisphenols may be relevant to the development of behavioural symptoms in childhood with sex-specific effects, although the results of existing studies are not consistent. The aim of the study was to evaluate the cross-sectional association between childhood exposure to these compounds and behavioral outcomes in the REPRO_PL cohort (Poland).

**Methods:**

Behavioral assessments were performed at the age of 7-9 years by parents using the Strengths and Difficulties Questionnaire (SDQ). HPLC–MS/MS was used for the quantification of BPA and 21 phthalate metabolites corresponding to 11 phthalate compounds (*n* = 400) and their replacement alternatives BPF, BPS, three metabolites of diethylhexyl terephthalate (DEHTP) and three metabolites of di-isononyl-cyclohexane-1,2-dicarboxylate (DINCH) (*n* = 150). Multivariable linear regression models accounting for sex-specific effects as well as sex-adjusted models were applied, using both separate models for each metabolite (or sum of metabolites) and joint models. In addition, mixtures models adjusted by the three chemical groups studied were also performed.

**Results:**

Median concentrations of several phthalate metabolites and bisphenols were of 42 µg/L (MEP), 4.5 µg/L (MMP), 3.5 µg/L (ΣDiDP), 2 µg/L (BPA) and 1 µg/L (BPF). For ΣDEHTP and ΣDINCH, the median concentrations were 35 µg/L and 3.1 µg/L, respectively. Exposure to phthalates was related to behavioral problems in girls, and bisphenols and DEHTP in boys. Among girls, DiBP was associated with mental health problems (total difficulties: β = 4.84; 95% CI 0.72;8.96, emotional: β = 2.14; 95% CI 0.33;4.0, hyperactivity/inattention: β = 2.52; 95% CI 0.55;4.49, externalizing behavior: (β = 2.95; 95% CI 0.36;5.53) and DiDP with hyperactivity/inattention scores (β = 2.46; 95% CI 0.30;4.63). BPF was associated with emotional problems and internalizing behavior among boys in both main and sensitivity models (main model: β = 1.03; 95% CI -0.16;2.21 and β = 1.71; 95% CI -0.14;3.56 respectively).

**Conclusions:**

This study shows that children’s exposure to several replacement compounds of BPA and phthalates, such as BPF and DEHTP, are associated with adverse effects on school-age children's behavior, with a divergent sex-specific effect. In any case, mixture models did not provide any further insight on the aforementioned cross-sectional associations and further methodological approaches are needed to explore adverse neurodevelopmental outcomes in children and teenagers.

**Supplementary Information:**

The online version contains supplementary material available at 10.1186/s12940-025-01210-6.

## Background

Endocrine Disrupting Chemicals (EDCs) are exogenous chemicals that represent public health concern. These substances, including phthalates and bisphenols, disrupt the physiological function of hormones by affecting their synthesis, secretion, transport, metabolism, receptor binding or elimination [33]. Widespread exposure to EDCs resulting from ingestion, inhalation and skin contact have been previously shown [6, 12, 25, 39, 58]. Phthalates are common in products used for personal hygiene, cosmetics, flooring materials and wallpapers, food packaging and children’s toys, among many others, due to their ability to enhance the flexibility and durability of plastics. Bisphenols are widely used in the production of plastics, resins, and thermal paper products.

Considering endocrine effects, several phthalates and BPA (4,4’-(propane-2,2-diyl)diphenol) have been progressively restricted in consumer product groups by national and international legislations and are subject to extensive regulations under REACH. In 1999, some restrictions were introduced on the content of six phthalates in toys and care products [10]. Di-2-ethylhexyl phthalate (DEHP), di-*n*-butyl phthalate (DnBP) and butylbenzyl phthalate (BBzP) were further restricted and regarding di-*iso*-nonyl phthalate (DiNP), di-*n*-octyl phthalate (DnOP), and di-*iso*-decyl phthalate (DiDP), only in cases where articles could be placed in the mouth [13]. DEHP, DnBP, BBzP and di-*iso*-butyl phthalate (DiBP) were finally restricted by the European Commission [11]. Moreover, the following phthalates: DEHP, DnBP, DiBP, BBzP (since 2015) and di-*n*-pentyl phthalate (DnPeP) (since 2020) require authorization to be placed or used [50]. The European Food Safety Authority (EFSA) has significantly lowered the tolerable daily intake (TDI) for BPA and its use in plastics and thermal paper has been progressively banned (i.e. BPA has been banned in infant feeding bottles across the EU since June 2011; in plastic bottles and packaging containing food for babies and children under three years since September 2018; in thermal paper receipts since January 2020; in food contact materials since December 2024) [15].

The aforementioned regulations have led to changes in phthalate and bisphenol exposure profile, phasing out by manufacturers and consequently decreasing exposures to DiBP, DnBP, BBzP, DEHP, BPA on the one hand and replacing them with analogues such as di-2-ethylhexyl terephthalate (DEHTP) and di-isononyl-cyclohexane-1,2-dicarboxylate (DINCH), bisphenol F (BPF, a mixture of isomeric congeners of 2,2'-, 2,4'- and 4,4'-dihydroxydiphenylmethane) and bisphenol S (BPS, 4,4'-sulfonylbisphenol) on the other [17, 30, 31, 35, 36, 58]. DEHTP and DINCH are used as alternatives of high molecular phthalates, including DEHP, while BPF is used in coatings and adhesives, plastic materials for dental sealants, water pipes and coatings for food packaging and is naturally present in mustard, whereas BPS is the major substitute in the production of thermal paper [7, 17]. For the bisphenols we can assume similar potential health effects, while the plasticizer substitutes DINCH and DEHTP seem to be considerably less potent in their reproductive toxicity [20, 35].

Young people are at increased risk of detrimental health effects posed by exposures to endocrine disrupting compounds given the ongoing endocrine processes that such substances can affect [43]. Prenatal and postnatal exposure to phthalates and bisphenols was indicated to be correlated with behavioral symptoms, including externalizing behavior (e.g., hyperactivity and aggressiveness) and internalizing behavior (e.g. anxiety, depression) with more consistent results observed for prenatal exposure [41]. Moreover, usually internalizing and externalizing symptoms seem to coexist and the associations usually, but not always, differ by sex. However, the results are still inconclusive [23]. There are few studies dedicated to BPS, BPF, DINCH and DEHTP and children's behaviors that examine the modification of effects by child sex [23, 42, 52].

We aimed to measure cross-sectional associations between childhood exposures to 11 phthalates, BPA as well as their replacement alternatives DEHTP, DINCH, BPF, and BPS, and behavioral outcomes in the REPRO_PL cohort (Poland). Advanced modeling based on a Bayesian approach accounting for sex-specific effects as well as sex-adjusted models were applied separately for each metabolite (or sum of metabolites) and jointly for all the chemical groups. In addition, a Bayesian mixture model was applied based on the three studied chemical groups (phthalates, non-phthalates and bisphenols).

## Methods

### Study design and population

This study is based on the REPRO_PL cohort, a longitudinal study in Poland, which has been described in previous publications [26, 45–47]. The REPRO_PL consists of the following phases covering key developmental periods: phase I—prenatal period (2007–2011), phase II—toddlerhood period (2008–2013), phase III—middle childhood period (2014–2019), phase IV—adolescence period (2022–2025). Out of 498 children who were examined at age of 7–9 years (phase III), samples of urine from 400 participants (80%) were used for the analysis of phthalates and BPA and from 150 participants (30%) for the analysis of DINCH, DEHTP, BPF and BPS. At the same time, information on outcome and covariates was collected. Accordingly, the analyses presented in this paper are based on a cross-sectional framework. The procedures within all 4 phases were approved by the Ethical Committee of NIOM, Lodz, Poland. In each case, written informed consents were received from the children's parents prior to the study.

## Exposure assessment

A spot urine sample was collected at age of 7–9 years and stored at −80ºC after aliquoting. All the analyses were performed at the Institute of the Ruhr-University Bochum (IPA), Germany and covered 21 metabolites equivalent to 11 phthalates (LOQs ranged between 0.2 µg/L and 1.0 µg/L), three metabolites of DEHTP (LOQ 0.2 µg/L), three metabolites of DINCH (LOQ 0.05 µg/L) and three bisphenols (BPA, BPF, BPS) (LOQ 0.25 µg/L; except for 250 samples on BPA, which LOQ was 0.10 µg/L). Briefly, 300 µl of urine were transferred into 1.8 mL vials, isotopically-labeled standards and 1 M ammonium acetate buffer were added (at different volumes, concentrations, and pH values, depending on the method employed [18, 20]). To hydrolyze possible glucuronide conjugated metabolites, 6 µL of β-glucuronidase from *E. coli* strain K-12 (arylsulfatase free) was used. The samples were mixed and incubated for 3–4 h at 37 ºC and then stored at −20 ºC overnight. After thawing and equilibration to room temperature, the samples were centrifuged for 10 min and the supernatant was transferred into a new HPLC vial for instrumental analysis. Biomarkers of phthalates, DINCH, DEHTP, and bisphenols were analysed using different online-SPE-HPLC–MS/MS methods with isotope dilution for quantification [19, 20, 29, 30, 35, 36, 54, 55]. The crude concentrations (µg/L) were also adjusted by creatinine concentrations (µg/g creatinine). The range was 0.10 – 1.69 g creatinine/L.

## Outcome assessment

During the visit at paediatric clinic (when the child was 7–9 years old) mothers were asked to fill in the SDQ questionnaire (Strengths and Difficulties Questionnaire). This tool is generally employed for the child behavioral assessment [22]. SDQ consists of one subscale measuring strengths (prosocial behaviour) and four subscales evaluating mental health problems (e.g. conduct, emotional, hyperactivity/inattention and peer relationship) [14]. Based on response categories for the five statements dedicated to each subscale (“not true” – 0, “somewhat true” – 1, and “certainly true”—2 points), a minimum of 0 points and a maximum of 10 points could be obtained. The sum of points from all subscales assessing mental health problems constituted the score on total difficulties (0–40 points). Score of the strength subscale was reversed prior to analysis so that for all SDQ subscales higher scores meant increased difficulties [44]. In addition to these five scales, internalizing and externalizing scores were calculated. For the internalizing score, two subscales were combined (e.g. emotional symptoms and peer relationship problems), while for the externalizing score, conduct problems and hyperactivity/inattention were summed. The number of points that could be obtained was 0–20 (the more points, the more problems).

## Covariates

A literature review was performed in order to identify potential confounders that may be associated with both exposure and child behavior. Socio-demographic information was provided by mothers during the visit at paediatric clinic. The analyses took into account the following variables: place of residence, maternal age, maternal education, number of siblings, socioeconomic status (SES), household status, traumatic events, child sex, child age and age at starting school. Environmental tobacco smoke (ETS) exposure at age of 7–9 years was assessed by cotinine levels in urine [38, 56]. Children's body mass index (BMI) categories were used as described by [32, 47].

## Statistical analysis

The statistical software R was used for statistical analysis [48]. Medians, percentiles and ranges were used for descriptive statistics on the pollutant concentrations, which were not normally distributed. Bayesian modeling was applied to account for the associations between behavioral outcomes and pollutant concentrations, using the arm package [21] for multivariable linear regression models and the BWQS package [9] for mixture models. In addition, an adapted version of the BayesGWQS package [59] was applied, because it allowed for the inclusion of different chemical families in the mixture models. The models focused on metabolites and compounds found in at least 90% of the analysed samples. In order to avoid amplification confounding bias [60], a directed acyclic graph (DAG) was built in order to identify potential confounders and at the same time to avoid causal intermediates or colliders (Figure S1). All multivariable linear models accounting for sex-specific effects were adjusted by maternal age, maternal education, household status, number of siblings, child’s age, BMI and urinary cotinine levels. In the models without sex-stratification, child sex was additionally added as a covariate. The sensitivity analyses were run with the following additional covariates included in the models: SES, age at starting school, traumatic events and place of residence, after removing the following variables previously included in the former models: maternal education, child’s age and household status. The pollutant concentrations were logarithmically transformed, and all continuous variables were scaled to two standard deviations. Additionally, joint models were performed with sex-adjusted and sex-stratified approaches, in which MMP, MEP, MBzP and sums of phthalate metabolites (ΣDiBP, ΣDnBP, ΣDEHP, ΣDiNP and ΣDiDP), sums of non-phthalate plasticizers metabolites (ΣDINCH and ΣDEHTP) and bisphenols (BPA and BPF) were included in the models together with the aforementioned covariates. For joint models, sensitive analyses were also performed, using the same previously reported covariates. In addition to linear models, where the outcomes are assumed to be linearly associated with the concentrations and the covariates, negative binomial models, in which the outcomes are considered as counts, were also performed. Comparisons between linear and negative binomial models were also performed (Figure S2). Given the similarities between the results of both types of models, only the linear ones are reported. Mixture models were based on a Bayesian Weighted Quantile Sum Regression (WQSR) approach, and were built clustering all the compounds together (for the basis mixture models) and grouping the compounds in three different groups, namely phthalates (3 individual metabolites and 5 sums of metabolites), non-phthalate plasticizers (2 sums of metabolites) and bisphenols (2 individual compounds), using the Bayesian Grouped WQSR. In the latter approach, we also checked for additional grouping of chemicals, including 2 groups (depending on their replacement nature, that is, phthalate metabolites and BPA in the first group, and non-phthalate metabolites and BPF in the second group) and 3 groups in a different approach than the previously mentioned (phthalate metabolites in the first group, BPA in the second group, and replacement alternatives –non-phthalate metabolites and BPF– in the third group). For this purpose, an adapted version for linear and negative binomial outcomes of the aforementioned package was used (data not shown). Four quantiles were selected in all the analyses, although we also checked the results for different quantiles, and the arguments for the fits were left as default. In order to make all the models comparable, the same set of covariates were included (see above). For all the figures, highest posterior densities of the beta-coefficients and their 95% credible intervals (CI; in some cases, also 90% CI), are reported.

## Results

### Characteristics of the population under study

The description of the participants can be found in Table 1. Briefly, most of the children lived in urban areas (≥ 75%) with both parents (> 85%). The largest number of mothers graduated from university (> 65%) and declared affluent or most affluent SES (> 90%). About 70% of respondents had at least one sibling. In accordance with the system of education in Poland, the children started education at the age of 6 (49%) or 7 (51%) years old. One-third of children (data for 383 children) were exposed to passive smoking. About 20% of participants were overweight or obese and 7% were classified as underweight. According to mothers’ declaration, about 15% of the children experienced traumatic events.

**Table 1:** Characteristics of the study population

Covariate	Total sample Phthalates and BPA *N* = 400 (%)	Subsample All chemicals (including substitutes) *n* = 150 (%)
Children’s sex		
Female	201 (50.2)	67 (44.7)
Male	199 (49.8)	83 (55.3)
Children’s age*		
7 years old	338 (84.5)	91 (60.7)
≥8 years old	62 (15.5)	59 (39.3)
Age at starting school^a^		
6 years	191 (48.7)	63 (43.4)
7 years	201 (51.3)	82 (56.6)
Place of residence		
Urban	329 (82.3)	113 (75.3)
Rural	71 (17.7)	37 (24.7)
Socio-economic status^a^		
Least affluent/affluent	303 (76.1)	103 (69.6)
Most affluent	95 (23.9)	45 (30.4)
Maternal age at child birth		
≤ 30 years	232 (58.0)	77 (51.3)
> 30 years	168 ( 42.0)	73 (48.7)
Maternal education^a^		
≤ 12	128 (32.2)	40 (27.0)
> 12	270 (67.8)	108 (73.0)
Household status^a^		
Both parents	346 (88.3)	133 (91.7)
Single parent	46 (11.7)	12 (8.3)
Number of siblings		
None	120 (30.2)	42 (28.2)
≥ 1	278 (69.8)	107 (71.8)
Cotinine level in child urine^a^*		
≤ 2.1 ng/ml	270 (70.5)	122 (91.0)
> 2.1 ng/ml	113 (29.5)	12 (9.0)
BMI groups		
Underweight	26 (6.5)	11 (7.3)
Recommended weight	296 (74.0)	104 (69.3)
Overweight/Obese	78 (19.5)	35 (23.3)
Traumatic events		
No	339 (86.5)	121 (84.0)
Yes	53 (13.5)	23(16.0)

^a^ Percentages calculated for observed values

^*^p < 0.05 – for comparison between total sample and subsample

## Characteristics of the exposure

**Fig. 1:**
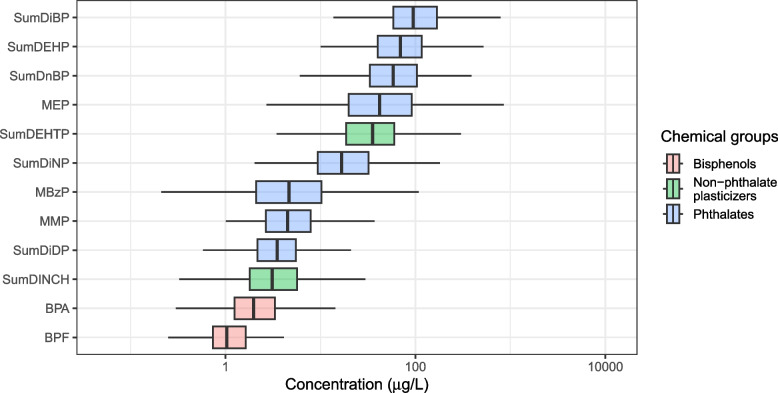
Boxplots showing the distribution of the concentrations (in µg/L) of metabolites of phthalate and non-phthalate plasticizers and bisphenols in 7–9-year-old children from Poland (n = 400 in the case of phthalate metabolites and BPA; *n* = 150 in the case of DINCH and DEHTP metabolites, and BPF)

Details regarding exposure to phthalates and bisphenols on a subpopulation of REPRO_PL 7–9-year-old children have been reported previously [18, 20], whereas the crude concentrations (µg/L) and creatinine-adjusted (µg/g creatinine) for metabolites of phthalates (*n* = 400), DINCH and DEHTP (*n* = 150), BPA (*n* = 399), BPF (*n* = 147) and BPS (*n* = 150) used in current analyses are shown in Table S1. The distribution of the concentrations of the chemicals is presented in Fig. 1. Seventeen phthalate metabolites were quantifiable in ≥ 95% (ten in 100% of the samples), and detection frequency (DF) for oxo-MiDP was 89%. MnPeP, MCHP and MnOP were found in 10% or less of the samples and were excluded from the main analyses. We noted the highest median levels for MiBP, MnBP and MEP, metabolites that belong to the LMW phthalates. DINCH and DEHTP metabolites were quantifiable in almost all samples (DF ≥ 97%) with median range from 0.6 µg/L (oxo-MINCH) to 1.6 µg/L (OH-MINCH), and from 2.7 µg/L (oxo-MEHTP) to 27 µg/L (cx-MEHTP). Overall, the median concentrations of ΣDEHTP (35 µg/L) and ΣDINCH (3.1 µg/L) were in the same range of phthalates such as MEP (42 µg/L) and ΣDiDP (3.5 µg/L) or MMP (4.5 µg/L), respectively. BPA was the most abundant bisphenol, quantifiable in 398 out of 399 samples (median 2.0 µg/L), followed by BPF (95% of the samples > LOQ, median 1.0 µg/L) and BPS (20% of the samples > LOQ, thus BPS was excluded from main analyses).

## Characteristics of the outcome

The SDQ scores are presented in Table 2. One in six participants fell within borderline/clinical range for the total difficulties score. Conduct problems were noted in 29% of the children, while about 20% of the children had peer relationship problems, hyperactivity/inattention or emotional symptoms. The scores obtained for the prosocial behaviour scale were within borderline and clinical range for about 7% of the children. The average number of points obtained for internalizing and externalizing behavior does not indicate problems in this area.

**Table 2:** Characteristics of behavioral outcomes

Behavioral outcomes	Mean (± SD)	Range	Borderline/Clinical
Conduct problems (0–10 points)	1.7 (± 1.4)^a^	0–7	29%^a^
	1.7 (± 1.4)^b^	0–6	29%^b^
Emotional symptoms (0–10 points)	2.2 (± 1.9)^a^	0–9	22%^a^
	2.0 (± 1.8)^b^	0–9	17%^b^
Hyperactivity/Inattention problems (0–10 points)	3.9 (± 2.4)^a^	0–10	24%^a^
	4.0 (± 2.3)^b^	0–9	23%^b^
Peer relationship problems (0–10 points)	1.4 (± 1.5)^a^	0–7	18%^a^
	1.3 (± 1.4)^b^	0–6	18%^b^
Total difficulties (0–40 points)	9.1 (± 5)^a^	0–26	17%^a^
	9.0 (± 4.9)^b^	0–25	15%^b^
Prosocial behavior (0–10 points)	8.4 (± 1.6)^a^	3–10	7%^a^
	8.5 (± 1.6)^b^	3–10	6%^b^
Internalizing scores (0–20 points)	3.5 (± 2.8)^a^	0–13	N/A
	3.3 (± 2.7)^b^	0–12	N/A
Externalizing scores (0–20 points)	5.6 (± 3.3)^a^	0–15	N/A
	5.7 (± 3.1)^b^	0–15	N/A

^a^ Data reported is based on 394 children with complete SDQ (Strengths and Difficulties Questionnaire) examination.^b^ Data reported is based on 147 children with complete SDQ (Strengths and Difficulties Questionnaire) examination

For each scale the following cut-offs are defined: Conduct problems as well as Peer relationships problems: 0–2 = normal, 3 = borderline, 4–10 = clinical; Emotional symptoms: 0–3 = normal, 4 = borderline, 5–10 = clinical; Hyperactivity/Inattention problems: 0–5 = normal, 6 = borderline, 7–10 = clinical; Prosocial behavior: 6–10 = normal, 5 = borderline, 0–4 = clinical; Total difficulties: 0–13 = normal, 14–16 = borderline, and 17–40 = clinical

## Cross-sectional associations between exposure to analysed chemicals and behavioral outcomes – single-pollutant models

**Fig. 2:**
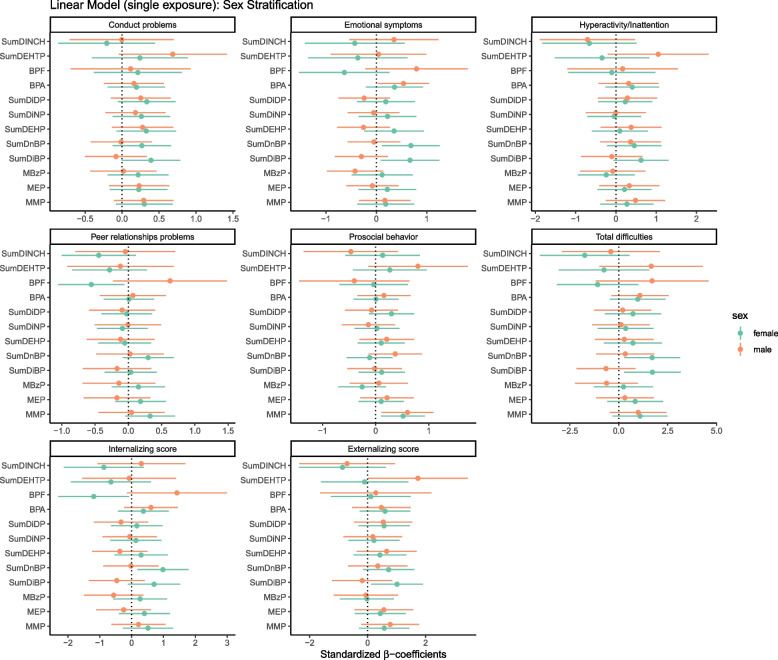
Standardized beta-coefficients (and 95% credible intervals) from single-pollutant multivariable linear regression models for phthalates, DINCH, DEHTP metabolites and bisphenols on the behavioral scales in children at 7–9 years of age (single-pollutant sex-stratified: One model is performed for each compound and each sex). Models are adjusted for maternal age at childbirth, maternal education, household status, number of siblings, child age, child BMI and cotinine levels in child urine.

The univariate linear regressions for SDQ and phthalates (MMP, MEP, MBzP, ΣDEHP, ΣDiDP, ΣDiNP, ΣDiBP and ΣDnBP), phthalate substitutes (ΣDINCH and ΣDEHTP) and bisphenols (BPA and BPF) for boys and girls are shown in Figure S4, while the results of multivariable linear regression models are posted in Fig. 2 and Tables S2 (main models) as well as Table S8 (sensitivity analyses). The results of sex-adjusted models can be found in Tables S3 (main model) and S9 (sensitivity analyses).

For both sexes, MMP was associated with increased prosocial behavior scores, indicating more difficulties (girls: β = 0.52; 95% CI 0.10;0.93; boys: β = 0.60; 95% CI 0.11;1.1) (Fig. 2, Table S2). The other associations between phthalates and behavioral outcomes were observed only for girls. DiBP and DnBP were associated with total difficulties (β = 1.7; 95% CI 0.27;3.2, β = 1.7; 95% CI 0.28;3.1, respectively) and emotional problems (β = 0.67; 95% CI 0.087;1.3, β = 0.69; 95% CI 0.11;1.3, respectively), DiBP with increased externalizing and internalizing scores (β = 1.0; 95% CI 0.12;1.9, β = 0.70; 95% CI −0.11;1.52, respectively), DnBP with increased internalizing scores (β = 0.98; 95% CI 0.18;1.8). Moreover, a positive trend was observed for DiBP and conduct as well as hyperactivity/inattention problems. In sex-adjusted models MMP was associated with total difficulties, conduct problems, prosocial behavior and externalizing scores, DnBP with total difficulties, DEHP and DiDP with conduct problems (Tables S3). Sensitivity analyses confirmed the results observed in the main models (Tables S8 and S9).

Higher DEHTP exposures were associated with conduct and externalizing problems among boys (β = 0.69; 95% CI −0.047;1.4; β = 1.7; 95% CI 0.017;3.5, respectively) (Fig. 2 and Table S2). In the sex adjusted models, DEHTP was associated with conduct problems and prosocial behavior (β = 0.42; 95% CI −0.043;0.88, β = 0.70; 95% CI 0.14;1.3, respectively) (Table S3). The results were confirmed in sensitivity analyses (Tables S8 and S9).

BPA was associated with emotional problems in boys (β = 0.54; 95% CI 0.032;1.1) (Fig. 2, Table S2). BPF was associated with decreased peer relationship scores in girls (β = −0.56; 95% CI −1.1;−0.061) and increased internalizing scores in boys (β = 1.4; 95% CI −0.14;3.0). In the sex-adjusted model for BPA, the coefficients for total difficulties and emotional problems were statistically significant (β = 1.0; 95% CI -0.0050;2.0 and β = 0.44; 95% CI 0.058;0.82, respectively). The results were afterwards confirmed in sensitivity analysis (Tables S8 and S9).

## Cross-sectional associations between the exposure to analyzed chemicals and behavioral outcomes – Joint exposure linear regression models and mixture models

**Fig. 3:**
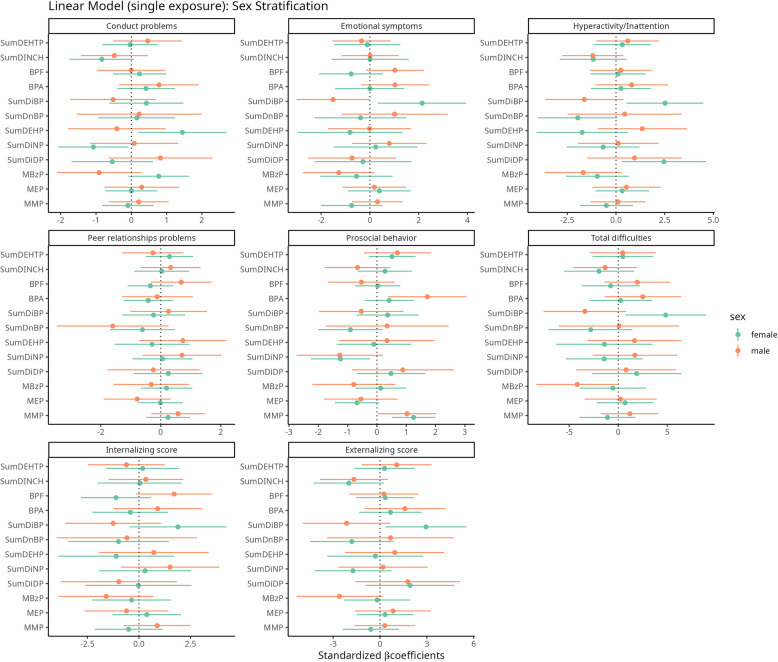
Standardized beta-coefficients (and 95% credible intervals) from joint multivariable linear regression models for phthalates, DINCH, DEHTP metabolites and bisphenols on the behavioral scales in children at 7–9 years of age (joint-pollutant sex-stratified: One model is performed for each sex accounting for all chemical compounds). Models are adjusted for maternal age at childbirth, maternal education, household status, number of siblings, child age, child BMI and cotinine level in child urine

The results of the joint models adjusted by all the chemical groups (either individual metabolites (e.g. MMP, MEP, MBzP) or sums of metabolites from common parent phthalates or non-phthalate plasticizers, aside of BPA and BPF) are presented in Fig. 3 and Tables S4 and S5 (main models) and Tables S10-S11 (sensitivity analyses). Several phthalates were associated with SDQ scores at 7–9 years, with some differences by sex (Fig. 3, Table S4). Higher concentrations of MMP were associated with prosocial behavior scores for both sexes (girls: β = 1.3; 95% CI 0.51;2.0; boys: β = 1.0; 95% CI 0.028;2.0). DiBP was associated with mental health problems in girls (total difficulties: β = 4.8; 95% CI 0.72;9.0, emotional: β = 2.1; 95% CI 0.33;4.0, hyperactivity/inattention: β = 2.5; 95% CI 0.55;4.5, externalizing behavior: β = 2.9; 95% CI 0.36;5.5) (Fig. 3, Tables S4). Among girls, DiNP was associated with decreased conduct and prosocial behavior scores (β = −1.1; 95% CI −2.1;−0.076, β = −1.3; 95% CI −2.3;−0.23), DiDP with increased hyperactivity/inattention scores (β = 2.5; 95% CI 0.30;4.6). The above associations were confirmed by the sensitivity models (Table S10). In sex-adjusted models DEP, BBzP, DiNP were associated with decreased SDQ scores (prosocial behavior, hyperactivity/inattention, prosocial behavior; respectively) and DMP, DiBP and DiDP with increased SDQ scores (prosocial behavior, externalizing, hyperactivity/inattention; respectively) (Tables S5 and S11).

No associations between DINCH and DEHTP and any child behavioral outcomes were observed that are consistent in the two (main and sensitivity) sex-stratified models (Fig. 3, Tables S4 and S10). In sex-adjusted models DINCH was associated with decreased hyperactivity/inattention scores (β = −1.2; 95% CI −2.2;−0.10) and DEHTP with increased prosocial behavior scores (β = 0.80; 95% CI 0.16;1.4); confirmed in sensitivity models (Tables S5 and S11).

Exposure to BPA was associated with prosocial behavior in boys (β = 1.7; 95% CI 0.39;3.1), which was not confirmed in sensitivity analysis (Fig. 3, Tables S4 and S10). This association was observed in sex-adjusted model (β = 0.92; 95% CI 0.19;1.7) (Tables S5 and S11). BPF was related to emotional problems and internalizing behavior among boys in both main and sensitivity models (main model: β = 1.0; 95% CI −0.16;2.2 and β = 1.7; 95% CI −0.14;3.6 respectively) (Fig. 3, Tables S4 and S10). Sex-adjusted models did not produce any significant results (Tables S5 and S11).

**Fig. 4:**
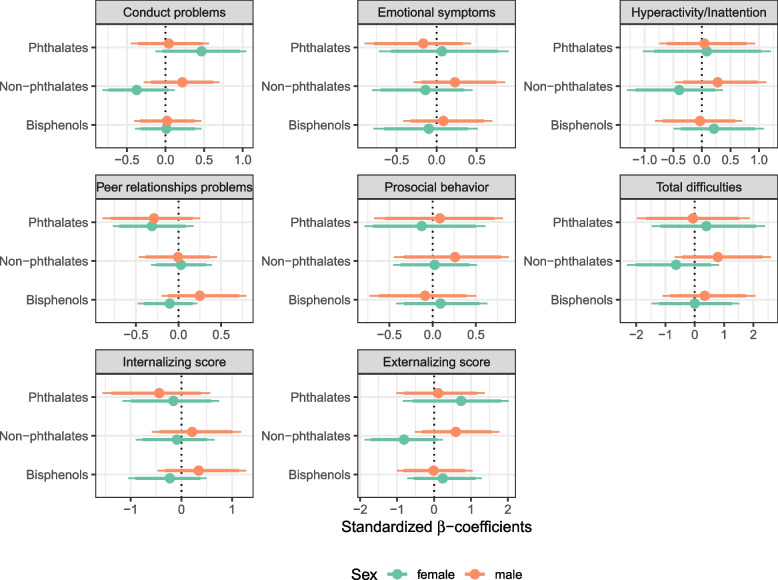
Standardized beta-coefficients (and 90% and 95% credible intervals) from mixture (grouped weighted quantile sum) regression models for three chemical groups (metabolites of phthalates (i), metabolites of non-phthalate plasticizers (i), and BPA and BPF (iii), respectively) on the behavioral scales in children at 7–9 years of age (mixture sex-stratified: One model is performed for each sex, accounting for the three chemical groups). Models are adjusted for maternal age at childbirth, maternal education, household status, number of siblings, child age, child BM and cotinine levels in child urine

Some of the aforementioned results were confirmed by weighted quantile sum regression models using three chemical groups (Fig. 4 and Tables S6 and S7). Specifically, exposures to phthalates were positively associated with conduct problems in girls, while the non-phthalate plasticizers showed a negative trend with conduct problems and externalizing behavior among girls (these cross-sectional associations were only statistically significant at 90%). Alternative mixed models with three different groups (including BPF in the non-phthalate plasticizers, to make a whole group of phthalates and BPA substitutes), provided similar results (data not shown). This alternative mixed model also confirmed previous results found in joint sex-adjusted models, including a positive association between exposures to BPA and higher prosocial behavior scores (data not shown).

In any case, mixture models did not provide any further insight on the aforementioned cross-sectional associations, since all the statistical significance found was at a 90% credible interval level (Fig. 4, Tables S6 and S7). Only in the case of the mixture models accounting for a single family including all chemical compounds in the same group provided a decreased peer relationships scores in girls, with statistical significant results in both model approaches tested (Figure S3).

## Discussion

The study has shown that children’s exposures to BPA and several phthalates as well as their replacement compounds are cross-sectionally associated with behavioral outcomes, with a divergent sex-specific effect. More precisely, exposure to phthalates was related to behavioral problems in girls, and bisphenols and DEHTP in boys.

This study presents a comprehensive group of metabolites of plastic-related additives such as phthalates and non-phthalate alternatives as well as bisphenols determined in Polish children. Exposure levels (for phthalates and bisphenols) for the REPRO_PL cohort were discussed in earlier published papers [18, 27]. A recently published paper by Vogel et al. [58] presents concentrations of phthalates/their metabolites (MEP, MBzP, MiBP, MnBP, ∑DEHP, ∑DiDP, ∑DiNP) and DINCH in European children aged 6–11 years (including Polish population) as results from HBM4EU 2021–2024 period Aligned Studies. As in our study, the metabolites of all the above-mentioned compounds had a detection frequency of almost 100%, which means that despite current legislation, phthalates are still prevalent, and that children are exposed to both phthalates and DINCH on a regular basis. As indicated by Vogel et al. [58], children in Denmark, The Netherlands and Hungary had the lowest concentrations across all metabolites compared to a European average, while children from Italy, France and Greece showed the highest (in Poland, low concentrations were noted for MBzP, ∑DINCH and ∑DiDP, while high for MEP, MnBP and ∑DEHP – which is in agreement with data from our study). In the REPRO_PL cohort, median concentrations were 1.6 µg/L for OH-MINCH and 0.91 µg/L for cx-MINCH compared to 2.2 µg/L for OH-MINCH and 1.2 µg/L for cx-MINCH in Vogel et al. [58] assessments (for all countries). BPA and its substitutes, BPF and BPS, are the most commonly analyzed in human biomonitoring studies. In the REPRO_PL cohort, BPA (median: 2.0 µg/L) and BPF (median: 1.0 µg/L) were quantifiable in almost all samples and BPS was found in 20% of the samples. Urinary BPA levels in REPRO_PL were similar to that observed in Brazil [51], higher compared to US-NHANES study [34] and Japan [24] and lower than in Norway [53], China [37], India [61] and US earlier study [5]. In REPRO_PL, median BPF level was higher than that noted in the Norway [53], US NHANES study [34], Brazil [51] and Japan [24]. It is not possible to compare the BPS levels observed in REPRO_PL with other studies, as most of the reported values were < LOQ. To conclude, differences in the level of exposure may result from children's characteristics (including child age), years of sampling and applicable regulations.

In our study, we found that children’s exposures to BPA and several phthalates as well as their replacement compounds are associated with behavioral outcomes, with a divergent sex-specific effect, which can be explained by the interaction between genetic background, hormones and environmental factors [41, 43]. The prenatal/postnatal bisphenols and phthalates exposures were found to be associated with behavioral symptoms in childhood [1, 4, 16, 40, 41, 43, 49, 62, 63]. It should be also pointed out that the majority of studies looking at sex-specific effects are focused on prenatal exposures to phthalates and BPA, and that there are limited number of studies for BPS, BPF, DINCH and DEHTP and children's behavior that examine the modification of sex [23, 42, 43, 52]. Prenatal exposure to phthalates increases internalizing and externalizing behaviors, as well as conduct problems in boys, but also in both sexes, as reported in some studies [43]. Prenatal BPA levels have been associated with higher externalizing behaviors and decreased anxiety in girls, but increased anxiety, emotional symptoms and internalizing behaviors in boys [43]. Although there is high heterogeneity in the studies, a recent systematic review and meta-analysis also reported that BPA exposures were associated with an increased risk of neurodevelopmental disorders and problems in children, particularly in boys [63]. Results of the study by Salamanca et al. [52] suggests that children’s exposure to DnBP and DEHP metabolites are associated with more externalizing problems in boys, higher exposures to DINCH may be associated with lower systemic brain-derived neurotrophic factor (BDNF) levels in boys and higher urinary BDNF concentrations may predict internalizing problems. Variations in findings between studies may be related to several reasons, including differences in set of compounds/metabolites being analyzed (and selected models for analyses), differences in time periods for exposure and outcome assessments, differences in exposure levels, differences in selected tools for children’s behavioral assessment and covariates included in analyses.

The main strength of the REPRO_PL study is related to the assessment of 21 phthalate metabolites corresponding to 11 phthalate compounds (18 finally included in analyses) and BPA in a total of 400 children, as well as their replacement alternatives, namely three metabolites of DEHTP, three metabolites of DINCH, BPF and BPS in 150 children. Although humans are exposed to a mixture of compounds the existing studies usually focus on single exposure or only several of them. Our analysis still have not covered global and comprehensive assessment (i.e., lead, cadmium, mercury and policyclic aromatic hydrocarbons were not considered in the current analyses), however it included a large group of chemical compounds and a diversity of multivariable regression models, accounting for each metabolite (or sum of metabolites) as well as a joint approach, adjusted by all the chemical groups, and a mixture model including the three chemical groups. Evaluation of child behavior was based on SDQ, frequently used and a valid tool. Sex specific associations, yet not always performed in other studies, is a strength of this study. Finally, various confounders (assessed prospectively and by biomarkers) were considered in the multivariable linear regression and in the grouped weighted quantile sum regression models.

Several limitations should be mentioned. The current evaluation has covered exposures at the age of 7–9 years, determined on a single occasion with exposure biomarkers that are rather rapidly excreted, and their impact on child behavior. For short lived chemicals, spot urine samples do have inherent variabilities due to their excretion kinetics, time point(s) of exposure and differences in hydration status. This might lead to some misclassifications especially of past or erratic exposures, however in general the bias would be towards underestimation rather than overestimation of associations [57]. Still, spot urine samples have generally been shown to characterize population distributions of exposures rather well, although caution should be exercised when interpreting data from low- or high-level spot samples [2, 3, 8]. An assessment of fetal and early life (age 2) exposures to specific phthalate metabolites was already conducted in a REPRO_PL sub-sample [27], as well as for phthalate metabolites and BPA (n = 250) during childhood [19, 28]. The joint and the mixture models taking into account all chemical compounds or chemical groups, respectively, were performed in a subset of 150 children, hence a decrease in sample size impedes comparability with separate models. Some associations could be due to chance (particularly those that appear in isolation not following a pattern of associations, and associations for new replacement chemicals with a lower weight of evidence). Also the mixture models are very sensitive to the chemical compounds included in each group. We observed that the combinations of compounds in different sets of groups produced different associations (data not shown). In any case, we decided to focus on the nature of the chemicals themselves to build the groups, namely phthalates, non-phthalate substitutes and bisphenols. Finally, despite the fact that the analyses included certain associated variables, the influence of other non-adjusted ones (i.e. Home Observation Measurement of the Environment (HOME), maternal anxiety/depressive symptoms, maternal IQ, satisfaction with overall living conditions) cannot be excluded.

## Conclusions

This study showed that not only phthalates and BPA, but also their increasingly used replacement alternatives such as DEHTP and BPF may be cross-sectionally associated with behavioral problems in children from Poland. Sex differences that were observed need to be marked. More mechanistic or toxicological evidence would be helpful for new replacement chemicals such as bisphenol analogues and DEHTP. Further research in this area and the results obtained can strengthen legislative, educational and intervention efforts to protect vulnerable populations.

## Supplementary Information

Supplementary Material 1: Appendix A. Supplementary data. Supplementary data to this article can be found online

## Data Availability

Data will be available on https://repod.icm.edu.pl. To facilitate reproducibility and reuse, the code used to perform the statistical analysis and calculations is available at GitHub (https://github.com/mercegari/Impact-of-Plastic-related-chemicals-on-Emotional-and-Behavioral-health).

## Abbreviations

DMP: Dimethyl phthalate
DEP: Diethyl phthalate
BBzP: Butylbenzyl phthalate
DnPeP: Di-*n*-pentyl phthalate
DCHP: Di-cyclohexyl phthalate
DiBP: Di-*iso*-butyl phthalate
DnBP: Di-*n*-butyl phthalate
DEHP: Di-2-ethylhexyl phthalate
DiNP: Di-*iso*-nonyl phthalate
DiDP: Di-*iso*-decyl phthalate
DnOP: Di-*n-*octyl phthalate
MMP: Mono-methyl phthalate
MEP: Mono-ethyl phthalate
MBzP: Mono-benzyl phthalate
MnPeP: Mono-*n*-pentyl phthalate
MCHP: Mono-cyclo-hexyl phthalate
MiBP: Mono-isobutyl phthalate
OH-MiBP: 2OH-mono-*iso*-butylphthalate
MnBP: Mono-*n*-butyl phthalate
OH-MnBP: 3OH-mono-*n*-butyl phthalate
MEHP: Mono-2-ethylhexyl phthalate
OH-MEHP: Mono-2-ethyl-5-hydroxyhexyl phthalate
oxo-MEHP: Mono-2-ethyl-5-oxo-hexyl phthalate
cx-MEPP: Mono-2-ethyl-5-carboxypentyl phthalate
OH-MiNP: 7-OH-mono-methyloctyl phthalate
oxo-MiNP: 7-Oxo-mono-methyloctyl phthalate
cx-MiNP: 7-Carboxy-mono-methylheptyl phthalate
OH-MiDP: 6-OH-mono-propylheptyl phthalate
oxo-MiDP: 6-Oxo-mono-propylheptyl phthalate
cx-MiDP: Mono-2–7-methyl-7-carboxyheptyl phthalate
MnOP: Mono-*n*-octyl phthalate
MCPP: Mono-3-carboxypropyl phthalate *(MCPP is a secondary metabolite of DnOP, but also metabolite of several HMW and LMW phthalates)*
ΣDiBP: Sum of di-*iso*-butyl phthalate metabolites
ΣDnBP: Sum of di-*n*-butyl phthalate metabolites
ΣDEHP: Sum of di-2-ethylhexyl phthalate metabolites
ΣDiNP: Sum of di-*iso*-nonyl phthalate metabolites
ΣDiDP: Sum of di-*iso*-decyl phthalate metabolites
DINCH: Di-isononyl cyclohexane-1,2-dicarboxylate
OH-MINCH: Cyclohexane-1,2-dicarboxylic acid-mono(hydroxyl-*iso*-nonyl) ester
oxo-MINCH: Cyclohexane-1,2-dicarboxylate-mono(oxo-isononyl) ester
cx-MINCH: Cyclohexane-1,2-dicarboxylate-mono-(7-carboxylate-4-methyl)heptyl ester
DEHTP: Di-2-ethylhexyl terephthalate
OH-MEHTP: Mono-(2-ethyl-5-hydroxy-hexyl) terephthalate
5oxo-MEHTP: Mono-(2-ethyl-5-oxo-hexyl) terephthalate
5cx-MEPTP: Mono-(2-ethyl-5-caboxyl-pentyl) terephthalate
BPA: Bisphenol A, 4,4’-(propane-2,2-diyl)diphenol)
BPF: Bisphenol F, a mixture of the isomeric congeners 2,2’-, 2,4’-, and 4,4’-dihydroxydiphenyl-methane
BPS: Bisphenol S, 4,4’- sulfonylbisphenol
SDQ: Strengths and Difficulties Questionnaire
WQSR: Weighted Quantile Sum Regression
