# Supplementary material for: Impact of plastic-related chemicals on emotional and behavioral health in children from Poland
Source: Environ Health. 2025 Oct 14;24:76. doi: 10.1186/s12940-025-01210-6 (PMC12523086; doi:10.1186/s12940-025-01210-6)

Impact of plastic-related chemicals on emotional and behavioral health in children from Poland

Kinga Polańska, Agnieszka Jankowska, Daniel Bury, Rebecca K. Moos, Claudia Pälmke, Joanna Jerzynska, Joanna Jurewicz, Stephan Bose-O'Reilly, Holger M. Koch, Mercè Garí

Supplementary data

Table S1. Concentrations for phthalate metabolites (n=400), metabolites of DINCH and DEHTP (n=150), BPA (n=399), BPS (n=150) and BPF (n=147) in the REPRO_PL cohort, in non-adjusted (µg/L) and creatinine-adjusted (µg/g creatinine) results

Table S2. Single-pollutant multivariable linear regression models applied on SDQ – Sex-stratified approach

Table S3. Single-pollutant multivariable linear regression models applied on SDQ – Sex-adjusted approach

Table S4. Joint-pollutant multivariable linear regression models applied on SDQ – Sex-stratified approach

Table S5. Joint-pollutant multivariable linear regression model applied on SDQ – Sex-adjusted approach

Table S6. Mixtures model Weighted Quantile Sums regression models applied on SDQ – Sex-stratified approach

Table S7. Mixtures model Weighted Quantile Sums regression model applied on SDQ – Sex-adjusted approach

Table S8. Single-pollutant multivariable linear regression models applied on SDQ with additional covariate adjustment – Sex-stratified approach

Table S9. Single-pollutant multivariable linear regression model applied on SDQ with additional covariate adjustment – Sex-adjusted approach

Table S10. Joint-pollutant or mixtures model with additional covariate adjustment – Sex-stratified approach

Table S11. Joint-pollutant or mixtures model with additional covariate adjustment – Sex-adjusted approach

Figure S1. Directed Acyclic Graph of potential confounders of the association between childhood phthalate, DINCH, DEHTP and bisphenols exposure and child behavior.

Figure S2. Correlation plots showing the beta-coefficients from multivariable linear *vs.* negative binomial regression models applied on SDQ. Panel A) Single-pollutant models for sex-adjusted and sex-stratified;Panel B) Joint-pollutant models for sex-adjusted and sex-stratified.

Figure S3. Comparison of Mixtures models from Grouped BayesGWQS for a single family *vs.* BWQS packages applied on SDQ in the sex-stratified approach.

Figure S4. Correlation plot showing the crude cross-sectional (univariate) associations between SDQ scales and concentrations of phthalate, ΣDINCH and ΣDEHTP metabolites and bisphenols (BPA and BPF), by sex.

Table S1. Concentrations for phthalate metabolites (n=400), metabolites of DINCH and DEHTP (n=150), BPA (n=399), BPS (n=150) and BPF (n=147) in the REPRO_PL cohort, in non-adjusted (μg/L) and creatinine-adjusted (μg/g creatinine) results

Compound	Metabolite	LOQ (μg/L)	DF (%)	Non-adjusted results (μg/L)					Creatinine adjusted results (μg/g creatinine)				
				Median	P25	P75	P90	Range	Median	P25	P75	P90	Range
DMP	MMP	1.0	96	4.5	2.7	7.9	15	<LOQ – 10500	6.5	3.8	11	21	<LOQ – 1167
DEP	MEP	0.50	100	42	20	91	159	2.7 – 1820	60	34	121	204	3.5 – 2861
BBzP	MBzP	0.20	98	4.7	2.1	10	21	<LOQ – 262	6.0	3.1	14	30	<LOQ – 804
DnPeP	MnPeP	0.20	5	<LOQ	<LOQ	<LOQ	<LOQ	<LOQ – 13	<LOQ	<LOQ	0.21	0.34	<LOQ – 13
DCHP	MCHP	0.20	6	<LOQ	<LOQ	<LOQ	<LOQ	<LOQ – 2.6	<LOQ	<LOQ	0.22	0.34	<LOQ – 3.6
DiBP	MiBP	1.0	100	69	43	130	196	1.8 – 2022	106	62	165	294	6.7 – 2727
	OH-MiBP	0.25	100	25	14	45	78	0.81 – 580	35	21	62	103	3.7 – 782
	ΣDiBP	-	-	95	58	169	273	2.6 – 2602	143	84	230	384	10.4 – 3509
DnBP	MnBP	1.0	100	51	28	91	148	1.8 – 8430	73	44	117	181	2.4 – 9367
	OH-MnBP	0.25	100	7.3	3.9	14	22	0.25 – 998	10	6.1	17	27	0.49 – 1109
	ΣDnBP	-	-	58	33	104	169	1.9 – 9428	81	50	134	211	2.9 – 10476
DEHP	MEHP	0.30	96	2.4	1.3	4.5	7.9	<LOQ – 625	3.2	1.8	6.9	12	<LOQ – 536
	OH-MEHP	0.20	100	23	13	40	64	0.97 – 4700	32	19	54	85	1.9 – 4029
	oxo-MEHP	0.20	100	17	10	30	48	0.69 – 3000	24	15	40	64	1.4 – 2571
	cx-MEPP	0.20	100	26	145	46	79	1.8 – 3350	38	22	63	104	1.9 – 2871
	ΣDEHP	-	-	44	25	73	116	1.8 - 8325	60	37	101	160	3.5 – 7136
DiNP	OH-MiNP	0.20	100	6.7	3.6	14	23	0.30 – 2430	9.8	5.0	18	31	0.48 – 2083
	oxo-MiNP	0.20	99	2.9	1.6	5.3	8.8	<LOQ – 1570	4.1	2.6	7.0	12	<LOQ – 1346
	cx-MiNP	0.20	100	6.6	3.7	12	21	0.43 – 1340	8.9	5.5	15	26	0.40 – 1149
	ΣDiNP	-	-	17	9.4	32	53	0.84 – 5340	23	14	41	66	0.95 – 4577
DiDP	OH-MiDP	0.20	96	1.7	1.0	2.7	5.3	<LOQ – 727	2.3	1.4	3.6	7.0	<LOQ – 623
	oxo-MiDP	0.20	89	0.90	0.47	1.6	3.1	<LOQ – 896	1.2	0.67	2.2	3.9	<LOQ – 768
	cx-MiDP	0.20	95	0.84	0.50	1.4	2.1	<LOQ – 77	1.2	0.77	1.9	2.8	<LOQ – 82
	ΣDiDP	-	-	3.5	2.2	5.5	11	0.30 – 1700	4.7	3.0	7.6	15	0.25 – 1457
DnOP	MnOP	0.20	1	<LOQ	<LOQ	<LOQ	<LOQ	<LOQ – 1.8	<LOQ	<LOQ	0.2	0.29	<LOQ – 2.1
Various	MCPP[a]	0.20	99	2.1	1.1	3.3	5.4	<LOQ – 142	2.8	1.7	4.3	6.8	<LOQ – 217
DINCH	OH-MINCH	0.050	100	1.6	0.96	3.1	7.1	0.090 – 36	2.2	1.6	3.8	8.2	0.53 – 49
	oxo-MINCH	0.050	99	0.62	0.41	1.2	2.5	<LOQ – 11	0.92	0.6	1.7	2.8	<LOQ – 14
	cx-MINCH	0.050	99	0.91	0.50	1.6	3.1	<LOQ – 15	1.2	0.8	2.1	3.7	<LOQ – 18
	ΣDINCH	-	-	3.1	1.8	5.7	13	0.21 - 62	4.3	3.1	7.0	14	1.1 – 81
DEHTP	OH-MEHTP	0.20	99	4.1	1.7	6.7	12	<LOQ – 33	5.3	2.9	8.7	13	<LOQ – 44
	oxo-MEHTP	0.20	97	2.7	1.3	5.4	10	<LOQ – 35	3.9	1.9	6.2	11	<LOQ – 48

	cx-MEPTP	0.20	100	27	15	51	86	3.0 - 939	39	21	68	121	4.4 – 1565
	ΣDEHTP	-	-	35	19	60	99	3.5 – 970	49	27	81	152	5.5 – 1617
BPA[b]		0.1/0.25	99	2.0	1.2	3.3	5.4	<LOQ - 53	2.8	1.8	4.7	9.4	<LOQ – 93
BPF		0.25	95	1.0	0.74	1.6	2.3	<LOQ - 90	1.3	1.0	1.8	2.7	<LOQ – 132
BPS		0.25	20	<LOQ	<LOQ	<LOQ	0.41	<LOQ – 7.0	0.19	0.14	0.35	0.63	<LOQ – 6.5

LOQ: Limit of Quantification;DF: Detection Frequency (Percentage above the LOQ);P25: Percentile 25th;P75: Percentile 75th;P95: Percentile 95th; DMP: Dimethyl phthalate;DEP: Diethyl phthalate;BBzP: Butylbenzyl phthalate;DnPeP: Di-*n*-pentyl phthalate;DCHP: Di-cyclohexyl phthalate;DiBP: Di-*iso*-butyl phthalate;DnBP: Di-*n*-butyl phthalate;DEHP: Di-2-ethylhexyl phthalate;DiNP: Di-*iso*-nonyl phthalate;DiDP: Di-*iso*-decyl phthalate;DnOP: Di-*n*-octyl phthalate;MMP: Mono-methyl phthalate;MEP: Mono-ethyl phthalate;MBzP: Mono-benzyl phthalate;MnPeP: Mono-*n*-pentyl phthalate;MCHP: Mono-cyclo-hexyl phthalate;MiBP: Mono-isobutyl phthalate;OH-MiBP: 2OH-mono-*iso*-butylphthalate;MnBP: Mono-*n*-butyl phthalate;OH-MnBP: 3OH-mono-*n*-butyl phthalate;MEHP: Mono-2-ethylhexyl phthalate;OH-MEHP: Mono-2-ethyl-5-hydroxyhexyl phthalate;oxo-MEHP: Mono-2-ethyl-5-oxo-hexyl phthalate;cx-MEPP: Mono-2-ethyl-5-carboxypentyl phthalate;OH-MiNP: 7-OH-mono-methyloctyl phthalate;oxo-MiNP: 7-oxo-mono-methyloctyl phthalate;cx-MiNP: 7-carboxy-mono-methylheptyl phthalate;OH-MiDP: 6-OH-mono-propylheptyl phthalate;oxo-MiDP: 6-oxo-mono-propylheptyl phthalate;cx-MiDP: Mono-2-7-methyl-7-carboxyheptyl phthalate;MnOP: Mono-*n*-octyl phthalate;MCPP: Mono-3-carboxypropyl phthalate *(MCPP is a secondary metabolite of DnOP, but also metabolite of several HMW and LMW phthalates);*ΣDiBP: sum of di-*iso*-butyl phthalate metabolites;ΣDnBP: sum of di-*n*-butyl phthalate metabolites;ΣDEHP: sum of di-2-ethylhexyl phthalate metabolites;ΣDiNP: sum of di-*iso*-nonyl phthalate metabolites;ΣDiDP: sum of di-*iso*-decyl phthalate metabolites;DINCH: Di-isononyl cyclohexane-1,2-dicarboxylate;OH-MINCH: Cyclohexane-1,2-dicarboxylic acid-mono(hydroxyl-*iso*-nonyl) ester;oxo-MINCH: Cyclohexane-1,2-dicarboxylate-mono(oxo-isononyl) ester;cx-MINCH: cyclohexane-1,2-dicarboxylate-mono-(7-carboxylate-4-methyl)heptyl ester;DEHTP: Di-2-ethylhexyl terephthalate;OH-MEHTP: Mono-(2-ethyl-5-hydroxy-hexyl) terephthalate;oxo-MEHTP: Mono-(2-ethyl-5-oxo-hexyl) terephthalate;cx-MEPTP: Mono-(2-ethyl-5-caboxyl-pentyl) terephthalate;BPA: Bisphenol A, 4,4'-(propane-2,2-diyl)diphenol);BPF: Bisphenol F, a mixture of the isomeric congeners 2,2'-, 2,4'-, and 4,4'-dihydroxydiphenyl-methane;BPS: Bisphenol S, 4,4'- sulfonylbisphenol

[a]MCPP is a metabolite of various LMW (DnBP) and HMW (DnOP, DiNP, DiDP) phthalates;[b] Two limits of quantification for BPA are reported, because the analysis was performed based on two methodologies. The first methodology analysed BPA alone, and had an LOQ of 0.10 µg/L (n=250 samples were analysed) while in the second methodology, BPA was analysed together with BPF and BPS, and the LOQ was 0.25 µg/L (n=149 samples were analysed).

Table S2. Single-pollutant multivariable linear regression models applied on SDQ – Sex-stratified approach

Compound	Metabolite	Sex	Total difficulties	Emotional symptoms	Conduct problems	Prosocial behavior	Hyperactivity/ Inattention problems	Peer relationships problems	Internalizing	Externalizing
			β-coeff (95% CI)							
DMP	MMP	Female	1.1 (-0.313;2.487)	0.186 (-0.378;0.75)	0.301 (-0.081;0.683)	**0.516 (0.103;0.929)****	0.272 (-0.387;0.931)	**0.328 (-0.045;0.7)***	0.514 (-0.274;1.301)	0.573 (-0.291;1.438)
		Male	0.994 (-0.455;2.444)	0.167 (-0.343;0.678)	0.293 (-0.11;0.696)	**0.598 (0.109;1.088)****	0.486 (-0.249;1.221)	0.048 (-0.448;0.545)	0.216 (-0.634;1.066)	0.779 (-0.221;1.778)
DEP	MEP	Female	0.838 (-0.587;2.262)	0.217 (-0.355;0.789)	0.225 (-0.164;0.614)	0.104 (-0.322;0.529)	0.212 (-0.458;0.881)	0.184 (-0.196;0.564)	0.401 (-0.4;1.201)	0.437 (-0.442;1.316)
		Male	0.312 (-1.158;1.783)	-0.079 (-0.595;0.437)	0.231 (-0.177;0.639)	0.214 (-0.288;0.716)	0.331 (-0.413;1.075)	-0.171 (-0.672;0.33)	-0.249 (-1.108;0.609)	0.562 (-0.45;1.574)
BBzP	MBzP	Female	0.243 (-1.267;1.753)	0.114 (-0.491;0.719)	0.217 (-0.195;0.628)	-0.255 (-0.703;0.193)	-0.24 (-0.947;0.467)	0.153 (-0.249;0.554)	0.267 (-0.58;1.113)	-0.024 (-0.955;0.907)
		Male	-0.623 (-2.226;0.979)	-0.423 (-0.982;0.137)	0.018 (-0.429;0.464)	0.06 (-0.488;0.608)	-0.078 (-0.891;0.736)	-0.141 (-0.688;0.406)	-0.564 (-1.497;0.369)	-0.06 (-1.167;1.048)
DiBP	ΣDiBP	Female	**1.718 (0.271;3.165)****	**0.667 (0.087;1.247)****	**0.39 (-0.007;0.787)***	0.113 (-0.324;0.55)	**0.624 (-0.058;1.306)***	0.037 (-0.355;0.428)	**0.704 (-0.114;1.522)***	**1.014 (0.121;1.907)****
		Male	-0.652 (-2.156;0.853)	-0.296 (-0.823;0.231)	-0.083 (-0.502;0.336)	-0.02 (-0.535;0.495)	-0.103 (-0.867;0.661)	-0.169 (-0.683;0.344)	-0.466 (-1.343;0.411)	-0.186 (-1.226;0.854)
DnBP	ΣDnBP	Female	**1.707 (0.279;3.135)****	**0.685 (0.113;1.257)****	0.268 (-0.126;0.662)	-0.113 (-0.544;0.318)	0.457 (-0.219;1.133)	0.298 (-0.086;0.682)	**0.983 (0.182;1.784)****	0.724 (-0.163;1.611)
		Male	0.331 (-1.153;1.815)	-0.046 (-0.567;0.474)	-0.013 (-0.426;0.4)	0.368 (-0.136;0.873)	0.364 (-0.387;1.114)	0.026 (-0.48;0.533)	-0.02 (-0.887;0.846)	0.351 (-0.673;1.374)
DEHP	ΣDEHP	Female	0.722 (-0.76;2.204)	0.351 (-0.242;0.944)	0.327 (-0.076;0.73)	0.105 (-0.337;0.547)	0.098 (-0.599;0.794)	-0.054 (-0.45;0.342)	0.298 (-0.535;1.131)	0.424 (-0.49;1.339)
		Male	0.284 (-1.213;1.78)	-0.255 (-0.778;0.269)	0.277 (-0.137;0.692)	0.208 (-0.303;0.718)	0.378 (-0.379;1.134)	-0.117 (-0.627;0.394)	-0.371 (-1.243;0.501)	0.655 (-0.374;1.683)
DiNP	ΣDiNP	Female	0.352 (-1.075;1.78)	0.222 (-0.349;0.794)	0.26 (-0.128;0.649)	0.025 (-0.4;0.45)	-0.043 (-0.712;0.627)	-0.088 (-0.469;0.292)	0.134 (-0.667;0.936)	0.218 (-0.662;1.098)
		Male	0.118 (-1.347;1.583)	-0.054 (-0.568;0.46)	0.181 (-0.226;0.588)	-0.138 (-0.638;0.362)	-0.004 (-0.746;0.738)	-0.005 (-0.505;0.494)	-0.059 (-0.914;0.796)	0.177 (-0.834;1.188)
DiDP	ΣDiDP	Female	0.729 (-0.704;2.163)	0.187 (-0.387;0.762)	**0.334 (-0.056;0.723)***	0.298 (-0.127;0.724)	0.233 (-0.44;0.905)	-0.024 (-0.407;0.359)	0.163 (-0.643;0.969)	0.566 (-0.316;1.449)
		Male	0.205 (-1.251;1.66)	-0.242 (-0.751;0.268)	0.253 (-0.151;0.656)	-0.077 (-0.574;0.42)	0.286 (-0.45;1.023)	-0.093 (-0.589;0.404)	-0.334 (-1.183;0.514)	0.539 (-0.463;1.541)

DINCH	ΣDINCH	Female	-1.739 (-4.024;0.546)	-0.428 (-1.417;0.561)	-0.207 (-0.861;0.447)	0.131 (-0.572;0.834)	-0.659 (-1.828;0.51)	-0.444 (-1.002;0.113)	-0.872 (-2.128;0.384)	-0.866 (-2.362;0.629)
		Male	-0.406 (-2.904;2.092)	0.349 (-0.535;1.232)	-0.005 (-0.708;0.698)	-0.468 (-1.356;0.421)	-0.705 (-1.886;0.476)	-0.044 (-0.793;0.705)	0.304 (-1.074;1.682)	-0.71 (-2.358;0.938)
DEHTP	ΣDEHTP	Female	-0.754 (-3.065;1.557)	-0.368 (-1.352;0.616)	0.241 (-0.408;0.89)	0.267 (-0.429;0.962)	-0.345 (-1.516;0.827)	-0.282 (-0.844;0.28)	-0.65 (-1.907;0.608)	-0.104 (-1.608;1.4)
		Male	1.659 (-0.98;4.299)	0.042 (-0.906;0.99)	**0.685 (-0.047;1.417)***	0.796 (-0.141;1.733)	1.05 (-0.199;2.298)	-0.118 (-0.918;0.681)	-0.076 (-1.551;1.398)	**1.735 (0.017;3.452)***
BPA		Female	0.97 (-0.448;2.388)	0.36 (-0.208;0.928)	0.194 (-0.194;0.583)	0.006 (-0.418;0.43)	0.407 (-0.258;1.072)	0.009 (-0.371;0.388)	0.369 (-0.429;1.167)	0.601 (-0.273;1.475)
		Male	1.083 (-0.373;2.538)	**0.539 (0.032;1.046)****	0.16 (-0.247;0.566)	0.156 (-0.343;0.655)	0.315 (-0.427;1.056)	0.069 (-0.426;0.564)	0.608 (-0.236;1.453)	0.474 (-0.535;1.483)
BPF		Female	-1.08 (-3.157;0.997)	-0.634 (-1.53;0.263)	0.213 (-0.38;0.806)	-0.038 (-0.683;0.607)	-0.103 (-1.184;0.978)	**-0.556 (-1.051;-0.061)****	**-1.19 (-2.3;-0.08)****	0.11 (-1.265;1.484)
		Male	1.706 (-1.174;4.585)	0.797 (-0.217;1.811)	0.115 (-0.696;0.927)	-0.402 (-1.44;0.636)	0.166 (-1.206;1.538)	0.627 (-0.227;1.482)	**1.424 (-0.143;2.991)***	0.282 (-1.643;2.207)

The models have been adjusted by maternal age at child birth, maternal education, household status, number of siblings, child age, child BMI, cotinine level in child urine. β-coefficient and 95% Confidence Intervals. ** p<0.05. * p<0.1

Table S3. Single-pollutant multivariable linear regression models applied on SDQ – sex-adjusted

Compound	Metabolite	Total difficulties	Emotional symptoms	Conduct problems	Prosocial behavior	Hyperactivity/Inattention problems	Peer relationships problems	Internalizing	Externalizing
		β-coeff (95% CI)							
DMP	MMP	**0.97 (-0.019;2.0)***	0.15 (-0.23;0.53)	**0.28 (0.0030;0.55)****	**0.53 (0.22;0.85)****	0.34 (-0.15;0.83)	0.21 (-0.098;0.51)	0.36 (-0.22;0.92)	**0.62 (-0.031;1.3)***
DEP	MEP	0.53 (-0.49;1.5)	0.051 (-0.33;0.43)	0.21 (-0.075;0.49)	0.14 (-0.19;0.47)	0.26 (-0.24;0.75)	0.011 (-0.30;0.32)	0.062 (-0.52;0.64)	0.47 (-0.20;1.1)
BBzP	MBzP	-0.19 (-1.3;0.90)	-0.13 (-0.54;0.28)	0.13 (-0.171;0.428)	-0.098 (-0.45;0.25)	-0.20 (-0.73;0.33)	0.018 (-0.31;0.35)	-0.11 (-0.73;0.51)	-0.072 (-0.78;0.63)
DiBP	ΣDiBP	0.50 (-0.53;1.5)	0.16 (-0.23;0.55)	0.15 (-0.136;0.438)	0.023 (-0.31;0.36)	0.27 (-0.23;0.78)	-0.077 (-0.39;0.24)	0.079 (-0.51;0.67)	0.42 (-0.25;1.1)
DnBP	ΣDnBP	**0.99 (-0.034;2.0)***	0.30 (-0.088;0.68)	0.13 (-0.159;0.41)	0.089 (-0.24;0.42)	0.41 (-0.085;0.91)	0.15 (-0.16;0.46)	0.45 (-0.14;1.0)	0.54 (-0.13;1.2)
DEHP	ΣDEHP	0.51 (-0.54;1.6)	0.054 (-0.34;0.45)	**0.29 (-0.001;0.577)***	0.14 (-0.19;0.48)	0.25 (-0.26;0.76)	-0.086 (-0.40;0.23)	-0.032 (-0.63;0.57)	0.54 (-0.14;1.2)
DiNP	ΣDiNP	0.21 (-0.81;1.2)	0.085 (-0.30;0.47)	0.20 (-0.076;0.485)	-0.038 (-0.36;0.29)	-0.043 (-0.54;0.45)	-0.040 (-0.35;0.27)	0.045 (-0.53 0.63)	0.16 (-0.50;0.82)
DiDP	ΣDiDP	0.40 (-0.61;1.4)	-0.057 (-0.44;0.33)	**0.28 (0.004;0.56)****	0.094 (-0.23;0.42)	0.25 (-0.25;0.74)	-0.069 (-0.38;0.24)	-0.13 (-0.71;0.45)	0.53 (-0.13;1.2)
DINCH	ΣDINCH	-0.99 (-2.7;0.70)	0.022 (-0.63;0.67)	-0.135 (-0.608;0.339)	-0.22 (-0.80;0.37)	-0.69 (-1.5;0.13)	-0.19 (-0.68;0.30)	-0.17 (-1.1;0.79)	-0.82 (-1.9;0.27)
DEHTP	ΣDEHTP	0.43 (-1.2;2.1)	-0.11 (-0.75;0.54)	**0.42 (-0.043;0.88)***	**0.70 (0.14;1.3)****	0.22 (-0.59;1.0)	-0.10 (-0.59;0.39)	-0.21 (-1.2;0.74)	0.64 (-0.45;1.7)
BPA		**1.0 (-0.005;2.0)***	**0.44 (0.058;0.82)****	0.18 (-0.097;0.46)	0.10 (-0.23;0.42)	0.34 (-0.15;0.83)	0.041 (-0.27;0.35)	0.48 (-0.096;1.1)	0.52 (-0.14;1.2)
BPF		0.64 (-1.1;2.4)	0.19 (-0.47;0.85)	0.14 (-0.34;0.61)	-0.14 (-0.74;0.46)	0.13 (-0.7;0.96)	0.19 (-0.31;0.69)	0.38 (-0.59;1.3)	0.27 (-0.85;1.4)

The models have been adjusted by maternal age at child birth, maternal education, household status, number of siblings, child age, child sex, child BMI, cotinine level in child urine. β-coefficient and 95% Confidence Intervals. ** $p<0.05$. * $p<0.1$

Table S4. Joint-pollutant multivariable linear regression models applied on SDQ – Sex-stratified approach

Compound	Metabolite	Sex	Total difficulties	Emotional symptoms	Conduct problems	Prosocial behavior	Hyperactivity/ Inattention problems	Peer relationships problems	Internalizing	Externalizing
			β-coeff (95% CI)							
DMP	MMP	Female	-1.094 (-3.955;1.767)	-0.746 (-2.006;0.514)	-0.098 (-0.827;0.632)	**1.251 (0.512;1.99)****	-0.497 (-1.864;0.871)	0.245 (-0.483;0.974)	-0.5 (-2.136;1.135)	-0.594 (-2.386;1.198)
		Male	1.192 (-1.734;4.117)	0.308 (-0.729;1.345)	0.208 (-0.637;1.053)	**1.026 (0.028;2.024)****	0.102 (-1.296;1.5)	0.576 (-0.312;1.463)	0.883 (-0.737;2.503)	0.31 (-1.628;2.248)
DEP	MEP	Female	0.702 (-2.199;3.602)	0.392 (-0.885;1.669)	0.004 (-0.736;0.743)	**-0.679 (-1.428;0.069)***	0.319 (-1.067;1.706)	-0.013 (-0.751;0.726)	0.379 (-1.279;2.038)	0.323 (-1.494;2.139)
		Male	0.232 (-3.437;3.901)	0.182 (-1.118;1.483)	0.296 (-0.763;1.356)	-0.545 (-1.797;0.706)	0.534 (-1.218;2.287)	-0.786 (-1.898;0.327)	-0.603 (-2.634;1.429)	0.832 (-1.598;3.261)
BBzP	MBzP	Female	-0.539 (-3.906;2.828)	-0.548 (-2.03;0.934)	**0.778 (-0.081;1.637)***	0.13 (-0.74;1)	-0.96 (-2.57;0.65)	0.194 (-0.663;1.051)	-0.354 (-2.277;1.57)	-0.183 (-2.293;1.926)
		Male	**-4.194 (-8.339;-0.05)***	**-1.273 (-2.742;0.196)***	-0.913 (-2.11;0.283)	-0.789 (-2.203;0.625)	-1.682 (-3.661;0.297)	-0.317 (-1.574;0.939)	-1.592 (-3.887;0.703)	**-2.597 (-5.342;0.147)***
DiBP	ΣDiBP	Female	**4.842 (0.722;8.962)****	**2.138 (0.326;3.95)****	0.428 (-0.624;1.479)	0.364 (-0.701;1.429)	**2.517 (0.547;4.488)****	-0.241 (-1.288;0.806)	1.894 (-0.458;4.245)	**2.945 (0.363;5.527)****
		Male	-3.398 (-7.604;0.808)	**-1.51 (-3.001;-0.019)***	-0.515 (-1.729;0.7)	-0.535 (-1.97;0.9)	-1.626 (-3.634;0.383)	0.26 (-1.015;1.536)	-1.252 (-3.581;1.077)	-2.142 (-4.927;0.643)
DnBP	ΣDnBP	Female	-2.799 (-7.059;1.461)	-0.381 (-2.255;1.492)	0.155 (-0.932;1.242)	-0.904 (-2.006;0.197)	**-1.96 (-3.998;0.078)***	-0.612 (-1.695;0.471)	-0.992 (-3.423;1.44)	-1.804 (-4.474;0.865)
		Male	0.089 (-6.043;6.221)	1.012 (-1.162;3.185)	0.22 (-1.549;1.989)	0.348 (-1.745;2.441)	0.446 (-2.478;3.371)	**-1.602 (-3.463;0.259)***	-0.586 (-3.983;2.811)	0.668 (-3.389;4.726)
DEHP	ΣDEHP	Female	-1.407 (-6.306;3.492)	-0.818 (-2.971;1.336)	**1.445 (0.193;2.697)****	-0.104 (-1.373;1.164)	-1.74 (-4.085;0.605)	-0.29 (-1.535;0.954)	-1.106 (-3.9;1.689)	-0.298 (-3.369;2.774)
		Male	1.66 (-3.134;6.453)	-0.017 (-1.716;1.682)	-0.409 (-1.793;0.975)	0.342 (-1.293;1.977)	1.348 (-0.941;3.637)	0.737 (-0.717;2.19)	0.719 (-1.935;3.374)	0.94 (-2.234;4.114)
DiNP	ΣDiNP	Female	-1.43 (-5.342;2.482)	0.238 (-1.484;1.959)	**-1.074 (-2.072;-0.076)****	**-1.245 (-2.255;-0.234)****	-0.66 (-2.53;1.211)	0.058 (-0.937;1.054)	0.295 (-1.939;2.53)	-1.732 (-4.183;0.719)
		Male	1.697 (-2.617;6.01)	0.796 (-0.733;2.325)	0.08 (-1.166;1.325)	**-1.268 (-2.74;0.203)***	0.113 (-1.948;2.173)	0.709 (-0.599;2.017)	1.506 (-0.883;3.894)	0.192 (-2.665;3.049)
DiDP	ΣDiDP	Female	1.885 (-2.64;6.41)	-0.282 (-2.272;1.708)	-0.54 (-1.695;0.615)	0.479 (-0.692;1.649)	**2.461 (0.295;4.626)****	0.252 (-0.898;1.402)	-0.03 (-2.612;2.553)	1.92 (-0.916;4.757)
		Male	0.8 (-4.275;5.875)	-0.731 (-2.53;1.068)	0.826 (-0.64;2.291)	0.881 (-0.851;2.613)	0.949 (-1.474;3.372)	-0.247 (-1.786;1.292)	-0.979 (-3.79;1.832)	1.775 (-1.585;5.136)
DINCH	ΣDINCH	Female	-1.956 (-5.542;1.63)	0.009 (-1.569;1.587)	**-0.835 (-1.75;0.079)***	0.274 (-0.652;1.2)	-1.167 (-2.882;0.547)	0.033 (-0.88;0.946)	0.041 (-2.007;2.09)	**-2.002 (-4.248;0.244)***
		Male	-1.339 (-4.614;1.935)	0.009 (-1.152;1.17)	-0.483 (-1.429;0.462)	-0.661 (-1.778;0.456)	-1.188 (-2.753;0.376)	0.329 (-0.663;1.322)	0.337 (-1.476;2.15)	-1.673 (-3.842;0.496)
DEHTP	ΣDEHTP	Female	0.469 (-2.619;3.558)	-0.112 (-1.471;1.247)	-0.033 (-0.82;0.755)	0.517 (-0.281;1.315)	0.321 (-1.155;1.798)	0.292 (-0.494;1.078)	0.179 (-1.585;1.944)	0.289 (-1.646;2.223)
		Male	0.46 (-2.902;3.822)	-0.345 (-1.537;0.847)	0.463 (-0.508;1.435)	0.696 (-0.451;1.843)	0.606 (-1.001;2.212)	-0.262 (-1.281;0.757)	-0.608 (-2.469;1.254)	1.069 (-1.159;3.296)

BPA	Female	0.251 (-2.955;3.456)	-0.003 (-1.414;1.409)	0.416 (-0.401;1.234)	0.416 (-0.412;1.243)	0.255 (-1.277;1.788)	-0.417 (-1.233;0.4)	-0.419 (-2.252;1.413)	0.671 (-1.336;2.679)
	Male	2.505 (-1.396;6.406)	1.026 (-0.357;2.409)	0.787 (-0.34;1.913)	**1.718 (0.387;3.049)****	0.804 (-1.059;2.668)	-0.116 (-1.299;1.067)	0.911 (-1.249;3.071)	1.592 (-0.993;4.176)
BPF	Female	-0.78 (-3.746;2.186)	-0.768 (-2.074;0.538)	0.233 (-0.523;0.989)	0.026 (-0.739;0.792)	0.104 (-1.313;1.521)	-0.347 (-1.102;0.408)	-1.114 (-2.81;0.581)	0.337 (-1.52;2.194)
	Male	1.955 (-1.385;5.294)	**1.027 (-0.157;2.21)***	-0.004 (-0.969;0.961)	-0.53 (-1.669;0.609)	0.25 (-1.346;1.845)	0.682 (-0.331;1.694)	**1.708 (-0.141;3.557)***	0.246 (-1.966;2.458)

The models have been adjusted by maternal age at child birth, maternal education, household status, number of siblings, child age, child BMI, cotinine level in child urine. β-coefficient and 95% Confidence Intervals. ** p<0.05. * p<0.1

Table S5. Joint-pollutant multivariable linear regression model applied on SDQ – Sex-adjusted approach

Compound	Metabolite	Total difficulties	Emotional symptoms	Conduct problems	Prosocial behavior	Hyperactivity/ Inattention problems	Peer relationships problems	Internalizing	Externalizing
		β-coeff (95% CI)							
DMP	MMP	0.139 (-1.836;2.115)	-0.132 (-0.904;0.641)	0.044 (-0.5;0.588)	**1.262 (0.666 ;1.859)****	-0.042 (-0.963;0.878)	0.269 (-0.292;0.831)	0.137 (-0.981;1.256)	0.002 (-1.242;1.246)
DEP	MEP	0.035 (-2.106;2.177)	0.171 (-0.666;1.009)	0.051 (-0.539;0.64)	**-0.699 (-1.345;-0.052)****	0.23 (-0.768;1.228)	-0.416 (-1.025;0.192)	-0.245 (-1.458;0.968)	0.281 (-1.068;1.629)
BBzP	MBzP	**-2.222 (-4.707;0.263)***	**-0.827 (-1.799;0.144)***	-0.028 (-0.712;0.657)	-0.142 (-0.892;0.609)	**-1.25 (-2.409;-0.092)****	-0.116 (-0.822;0.591)	-0.944 (-2.35;0.463)	-1.278 (-2.843;0.287)
DiBP	ΣDiBP	0.169 (-2.696;3.034)	0.049 (-1.07;1.169)	-0.062 (-0.851;0.727)	-0.066 (-0.932;0.8)	0.323 (-1.013;1.658)	-0.141 (-0.956;0.674)	-0.091 (-1.713;1.53)	**2.253 (0.234;4.271)****
DnBP	ΣDnBP	-0.92 (-4.188;2.348)	0.204 (-1.073;1.482)	0.122 (-0.777;1.022)	-0.697 (-1.685;0.29)	-0.628 (-2.151;0.896)	-0.62 (-1.55;0.309)	-0.415 (-2.265;1.435)	-1.059 (-2.807;0.69)
DEHP	ΣDEHP	1.075 (-2.3;4.45)	-0.077 (-1.396;1.242)	0.156 (-0.774;1.085)	0.175 (-0.845;1.195)	0.486 (-1.087;2.059)	0.51 (-0.45;1.47)	0.432 (-1.478;2.343)	0.642 (-1.483;2.767)
DiNP	ΣDiNP	-0.371 (-3.148 ;2.406)	0.413 (-0.672;1.499)	-0.463 (-1.227;0.302)	**-0.983 (-1.822;-0.144)****	-0.596 (-1.89;0.699)	0.273 (-0.516;1.063)	0.687 (-0.885;2.259)	-0.505 (-2.563;1.553)
DiDP	ΣDiDP	1.348 (-1.858;4.554)	-0.53 (-1.783;0.723)	0.435 (-0.448 ;1.318)	0.668 (-0.3;1.637)	**1.817 (0.322;3.311)****	-0.373 (-1.285;0.538)	-0.903 (-2.717;0.912)	0.26 (-1.544;2.065)
DINCH	ΣDINCH	-1.466 (-3.722;0.79)	0.115 (-0.767;0.997)	**-0.529 (-1.15 ;0.093)***	-0.275 (-0.957;0.406)	**-1.153 (-2.204;-0.101)****	0.101 (-0.541;0.742)	0.215 (-1.062;1.493)	0.997 (-0.516;2.51)
DEHTP	ΣDEHTP	0.051 (-2.073;2.176)	-0.282 (-1.113;0.548)	0.376 (-0.209;0.961)	**0.797 (0.155;1.438)****	0.006 (-0.984;0.996)	-0.049 (-0.653;0.555)	-0.331 (-1.534;0.872)	0.239 (-1.109;1.587)
BPA		0.95 (-1.453;3.354)	0.289 (-0.65;1.229)	0.466 (-0.195;1.128)	**0.92 (0.194;1.645)** **	0.531 (-0.59;1.651)	-0.337 (-1.02;0.347)	-0.047 (-1.407;1.314)	**-1.682 (-3.102;-0.261)** **
BPF		1.104 (-1.037;3.244)	0.372 (-0.465;1.209)	0.032 (-0.557;0.622)	-0.265 (-0.911;0.381)	0.207 (-0.791;1.204)	0.493 (-0.116;1.102)	0.865 (-0.347;2.077)	0.382 (-0.955;1.72)

The models have been adjusted by maternal age at child birth, maternal education, household status, number of siblings, child age, child sex, child BMI, cotinine level in child urine. β-coefficient and 95% Confidence Intervals. ** $p<0.05$. * $p<0.1$

Table S6. Grouped Weighted Quantile Sums regression models applied on SDQ – Sex-stratified approach

Compound	Sex	Total difficulties	Emotional symptoms	Conduct problems	Prosocial behavior	Hyperactivity/Inattention problems	Peer relationships problems	Internalizing	Externalizing
		β-coeff (95% CI)							
Phthalates	Female	0.395 (-1.47;2.393)	0.066 (-0.718;0.897)	0.466 (-0.13;1.046)	-0.126 (-0.784;0.61)	0.087 (-1.03;1.208)	-0.311 (-0.771;0.175)	-0.158 (-1.162;0.744)	0.732 (-0.842;2.021)
	Male	-0.062 (-1.969;1.874)	-0.167 (-0.897;0.43)	0.042 (-0.445;0.561)	0.084 (-0.676;0.811)	0.046 (-0.74;0.932)	-0.288 (-0.892;0.252)	-0.438 (-1.556;0.564)	0.115 (-1.015;1.375)
Phthalate substitutes	Female	-0.001 (-1.474;1.522)	-0.097 (-0.787;0.511)	0.01 (-0.394;0.466)	0.091 (-0.42;0.632)	0.211 (-0.491;1.084)	-0.104 (-0.476;0.219)	-0.228 (-1.049;0.49)	0.238 (-0.726;1.293)
	Male	0.341 (-1.096;2.065)	0.084 (-0.415;0.693)	0.02 (-0.405;0.462)	-0.087 (-0.732;0.503)	-0.033 (-0.817;0.705)	0.249 (-0.198;0.796)	0.34 (-0.469;1.281)	-0.011 (-1.005;1.041)
Bisphenols	Female	-0.64 (-2.312;0.825)	-0.14 (-0.805;0.446)	-0.375 (-0.816;0.118)	0.024 (-0.455;0.512)	-0.397 (-1.308;0.369)	0.029 (-0.322;0.388)	-0.088 (-0.9;0.652)	-0.813 (-1.875;0.242)
	Male	0.785 (-0.684;2.599)	0.228 (-0.287;0.853)	0.218 (-0.28;0.697)	0.259 (-0.446;0.878)	0.277 (-0.466;1.126)	-0.007 (-0.464;0.449)	0.212 (-0.581;1.174)	0.59 (-0.518;1.765)

The models have been adjusted by maternal age at child birth, maternal education, household status, number of siblings, child age, child BMI, cotinine level in child urine. β-coefficient and 95% Confidence Intervals.

Phthalates: MMP, MEP, MBzP, ΣDiBP, ΣDnBP, ΣDEHP, ΣDiNP and ΣDiNP; phthalate substitutes: ΣDINCH and ΣDEHTP; bisphenols: BPA and BPF.

Table S7. Grouped Weighted Quantile Sums regression model applied on SDQ – Sex-adjusted approach

Compound	Total difficulties	Emotional symptoms	Conduct problems	Prosocial behavior	Hyperactivity/Inattention problems	Peer relationships problems	Internalizing	Externalizing
	β-coeff (95% CI							
Phthalates	0.119 (-1.09;1.458)	-0.04 (-0.5;0.393)	0.094 (-0.219;0.483)	-0.224 (-0.756;0.583)	0.137 (-0.572;0.835)	-0.224 (-0.586;0.117)	-0.224 (-0.955;0.431)	0.301 (-0.551;1.254)
Phthalates substitutes	0.236 (-0.71;1.403)	0.026 (-0.353;0.438)	0.054 (-0.233;0.389)	0.217 (-0.324;0.67)	0.082 (-0.41;0.66)	0.044 (-0.233;0.359)	0.089 (-0.477;0.722)	0.16 (-0.507;0.961)
Bisphenols	-0.023 (-1.046;0.971)	0.044 (-0.33;0.453)	0.022 (-0.368;0.363)	0.23 (-0.361;0.601)	-0.174 (-0.747;0.328)	0.01 (-0.269;0.307)	0.042 (-0.461;0.627)	-0.225 (-1.039;0.567)

The models have been adjusted by maternal age at child birth, maternal education, household status, number of siblings, child age, child sex, child BMI, cotinine level in child urine. β-coefficient and 95% Credible Intervals.

Phthalates: MMP, MEP, MBzP, ΣDiBP, ΣDnBP, ΣDEHP, ΣDiNP and, ΣDiNP; phthalate substitutes: ΣDINCH and ΣDEHTP; bisphenols: BPA and BPF.

Table S8. Single-pollutant multivariable linear regression models applied on SDQ with additional covariate adjustment – Sex-stratified approach

Compound	Metabolite	Sex	Total difficulties	Emotional symptoms	Conduct problems	Prosocial behavior	Hyperactivity/ Inattention problems	Peer relationships problems	Internalizing	Externalizing
			β-coeff (95% CI)							
DMP	MMP	Female	1.011 (-0.395;2.416)	0.122 (-0.439;0.683)	0.294 (-0.091;0.68)	**0.487 (0.072;0.903)****	0.294 (-0.376;0.964)	0.3 (-0.069;0.669)	0.422 (-0.355;1.199)	0.589 (-0.291;1.469)
		Male	0.856 (-0.594;2.306)	0.101 (-0.408;0.611)	0.229 (-0.168;0.626)	**0.509 (0.019;0.998)****	0.464 (-0.277;1.205)	0.062 (-0.429;0.552)	0.163 (-0.683;1.009)	0.693 (-0.304;1.69)
DEP	MEP	Female	0.627 (-0.807;2.061)	0.11 (-0.461;0.681)	0.194 (-0.199;0.588)	0.043 (-0.385;0.472)	0.209 (-0.473;0.891)	0.114 (-0.263;0.491)	0.224 (-0.568;1.016)	0.403 (-0.494;1.3)
		Male	0.377 (-1.103;1.856)	-0.05 (-0.569;0.468)	0.226 (-0.178;0.63)	0.164 (-0.339;0.667)	0.387 (-0.367;1.142)	-0.187 (-0.685;0.312)	-0.237 (-1.097;0.623)	0.613 (-0.402;1.628)
BBzP	MBzP	Female	0.159 (-1.31;1.629)	0.041 (-0.543;0.625)	0.217 (-0.185;0.619)	-0.217 (-0.654;0.22)	-0.239 (-0.936;0.459)	0.139 (-0.246;0.525)	0.181 (-0.629;0.991)	-0.021 (-0.941;0.898)
		Male	-0.348 (-1.93;1.234)	-0.329 (-0.882;0.223)	0.002 (-0.431;0.436)	-0.051 (-0.589;0.487)	0.093 (-0.716;0.902)	-0.114 (-0.647;0.419)	-0.443 (-1.361;0.475)	0.095 (-0.995;1.185)
DiBP	ΣDiBP	Female	**1.939 (0.519;3.359)****	**0.704 (0.138;1.27)****	**0.467 (0.075;0.858)****	0.183 (-0.248;0.614)	**0.712 (0.032;1.392)****	0.056 (-0.324;0.437)	**0.761 (-0.03;1.551)***	**1.179 (0.29;2.068)****
		Male	-0.513 (-2.014;0.988)	-0.247 (-0.772;0.278)	-0.116 (-0.527;0.295)	-0.075 (-0.586;0.436)	0.012 (-0.757;0.78)	-0.162 (-0.668;0.344)	-0.409 (-1.281;0.463)	-0.104 (-1.139;0.931)
DnBP	ΣDnBP	Female	**1.676 (0.242;3.11)****	**0.654 (0.084;1.224)****	0.271 (-0.126;0.668)	-0.092 (-0.525;0.342)	0.488 (-0.199;1.175)	0.263 (-0.117;0.643)	**0.917 (0.126;1.708)****	0.759 (-0.144;1.663)
		Male	0.338 (-1.137;1.813)	-0.063 (-0.581;0.454)	-0.088 (-0.492;0.316)	0.297 (-0.203;0.797)	0.432 (-0.32;1.184)	0.057 (-0.44;0.555)	-0.006 (-0.864;0.852)	0.344 (-0.671;1.359)
DEHP	ΣDEHP	Female	0.68 (-0.779;2.139)	0.326 (-0.254;0.905)	**0.337 (-0.062;0.735)***	0.129 (-0.307;0.565)	0.073 (-0.621;0.768)	-0.056 (-0.44;0.329)	0.27 (-0.536;1.076)	0.41 (-0.503;1.323)
		Male	0.573 (-0.905;2.052)	-0.13 (-0.648;0.389)	0.258 (-0.146;0.662)	0.094 (-0.409;0.598)	0.566 (-0.187;1.318)	-0.12 (-0.619;0.378)	-0.25 (-1.111;0.61)	0.824 (-0.189;1.836)
DiNP	ΣDiNP	Female	0.505 (-0.909;1.918)	0.274 (-0.287;0.835)	0.309 (-0.077;0.695)	0.095 (-0.327;0.517)	-0.034 (-0.706;0.639)	-0.045 (-0.417;0.327)	0.229 (-0.551;1.01)	0.275 (-0.61;1.16)
		Male	0.306 (-1.163;1.776)	0.02 (-0.495;0.536)	0.155 (-0.246;0.557)	-0.137 (-0.637;0.362)	0.1 (-0.651;0.852)	0.031 (-0.465;0.526)	0.051 (-0.804;0.906)	0.256 (-0.756;1.267)
DiDP	ΣDiDP	Female	0.632 (-0.798;2.061)	0.159 (-0.41;0.728)	0.291 (-0.1;0.682)	0.301 (-0.124;0.726)	0.227 (-0.452;0.907)	-0.045 (-0.422;0.331)	0.113 (-0.676;0.903)	0.518 (-0.375;1.411)
		Male	0.251 (-1.214;1.717)	-0.223 (-0.736;0.289)	0.216 (-0.185;0.616)	-0.092 (-0.59;0.406)	0.328 (-0.42;1.076)	-0.069 (-0.563;0.425)	-0.292 (-1.143;0.559)	0.543 (-0.462;1.549)
DINCH	ΣDINCH	Female	-1.168 (-3.37;1.034)	-0.283 (-1.245;0.68)	-0.012 (-0.599;0.575)	0.138 (-0.599;0.876)	-0.499 (-1.654;0.656)	-0.374 (-0.946;0.198)	-0.657 (-1.918;0.604)	-0.511 (-1.926;0.904)
		Male	-0.221 (-2.745;2.303)	0.431 (-0.45;1.313)	0.118 (-0.565;0.801)	-0.522 (-1.394;0.349)	-0.714 (-1.926;0.499)	-0.056 (-0.846;0.734)	0.375 (-1.053;1.804)	-0.596 (-2.249;1.057)

DEHTP	ΣDEHTP	Female	-0.347 (-2.553;1.859)	-0.259 (-1.214;0.697)	0.307 (-0.268;0.883)	0.431 (-0.292;1.153)	-0.146 (-1.299;1.007)	-0.249 (-0.822;0.324)	-0.508 (-1.764;0.748)	0.161 (-1.249;1.572)
		Male	1.509 (-1.114;4.132)	-0.016 (-0.948;0.916)	**0.753 (0.061;1.445)****	**0.906 (0.009;1.803)***	0.99 (-0.273;2.253)	-0.218 (-1.046;0.61)	-0.235 (-1.737;1.267)	**1.743 (0.056;3.429)****
BPA		Female	0.84 (-0.581;2.262)	0.327 (-0.238;0.892)	0.142 (-0.249;0.532)	-0.016 (-0.441;0.41)	0.406 (-0.27;1.081)	-0.034 (-0.409;0.341)	0.293 (-0.493;1.079)	0.547 (-0.342;1.436)
		Male	0.848 (-0.581;2.277)	**0.464 (-0.034;0.961)***	0.185 (-0.206;0.577)	0.218 (-0.268;0.704)	0.14 (-0.595;0.874)	0.06 (-0.419;0.538)	0.523 (-0.302;1.348)	0.325 (-0.663;1.313)
BPF		Female	-0.645 (-2.652;1.361)	-0.364 (-1.244;0.516)	0.33 (-0.197;0.858)	-0.073 (-0.768;0.621)	-0.036 (-1.107;1.035)	**-0.575 (-1.092;-0.057)****	-0.939 (-2.072;0.194)	0.294 (-1.005;1.593)
		Male	**3.096 (0.49;5.702)****	**1.33 (0.433;2.226)****	0.291 (-0.443;1.026)	-0.117 (-1.078;0.844)	0.543 (-0.749;1.836)	**0.931 (0.103;1.758)****	**2.261 (0.823;3.699)****	0.835 (-0.951;2.62)

The models have been adjusted by maternal age at child birth, number of siblings, child BMI, cotinine level in child urine, child age at starting school, socio-economic status, place of residence and traumatic events. β-coefficient and 95% Confidence Intervals. ** p<0.05. * p<0.1

Table S9. Single-pollutant multivariable linear regression model applied on SDQ with additional covariate adjustment – Sex-adjusted approach

Compound	Metabolite	Total difficulties	Emotional symptoms	Conduct problems	Prosocial behavior	Hyperactivity/Inattention problems	Peer relationships problems	Internalizing	Externalizing
		β-coeff (95% CI)							
DMP	MMP	**0.885 (-0.11;1.88)***	0.107 (-0.267;0.482)	**0.259 (-0.017;0.535)***	**0.481 (0.165;0.797)****	0.338 (-0.152;0.827)	0.181 (-0.12;0.482)	0.288 (-0.276;0.853)	**0.597 (-0.057;1.251)***
DEP	MEP	0.506 (-0.508;1.52)	0.025 (-0.356;0.406)	0.206 (-0.075;0.488)	0.101 (-0.224;0.426)	0.283 (-0.215;0.781)	-0.008 (-0.315;0.298)	0.017 (-0.558;0.591)	0.489 (-0.176;1.155)
BBzP	MBzP	-0.075 (-1.129;0.979)	-0.112 (-0.508;0.283)	0.134 (-0.159;0.427)	-0.129 (-0.466;0.209)	-0.123 (-0.641;0.395)	0.026 (-0.292;0.345)	-0.086 (-0.683;0.511)	0.011 (-0.682;0.704)
DiBP	ΣDiBP	0.733 (-0.291;1.758)	0.214 (-0.17;0.599)	0.198 (-0.086;0.483)	0.048 (-0.281;0.377)	0.371 (-0.133;0.874)	-0.05 (-0.361;0.26)	0.164 (-0.417;0.745)	**0.569 (-0.104;1.242)***
DnBP	ΣDnBP	**1.034 (0.02;2.048)****	0.292 (-0.089;0.673)	0.133 (-0.15;0.416)	0.063 (-0.264;0.39)	**0.463 (-0.036;0.962)***	0.146 (-0.162;0.454)	0.438 (-0.138;1.014)	**0.596 (-0.072;1.264)***
DEHP	ΣDEHP	0.653 (-0.375;1.68)	0.096 (-0.29;0.482)	**0.299 (0.015;0.583)****	0.132 (-0.198;0.461)	0.32 (-0.185;0.825)	-0.062 (-0.373;0.249)	0.033 (-0.549;0.616)	**0.619 (-0.055;1.293)***
DiNP	ΣDiNP	0.431 (-0.58;1.443)	0.158 (-0.221;0.537)	**0.24 (-0.041;0.52)***	-0.007 (-0.331;0.318)	0.026 (-0.472;0.523)	0.008 (-0.298;0.314)	0.166 (-0.407;0.739)	0.265 (-0.4;0.93)
DiDP	ΣDiDP	0.499 (-0.513;1.51)	-0.028 (-0.408;0.351)	**0.299 (0.019;0.578)****	0.106 (-0.218;0.431)	0.284 (-0.212;0.781)	-0.056 (-0.362;0.25)	-0.084 (-0.658;0.489)	**0.583 (-0.08;1.246)***
DINCH	ΣDINCH	-0.879 (-2.567;0.809)	0.01 (-0.641;0.66)	-0.073 (-0.531;0.385)	-0.173 (-0.75;0.403)	-0.64 (-1.462;0.182)	-0.176 (-0.67;0.318)	-0.166 (-1.125;0.792)	-0.713 (-1.8;0.374)
DEHTP	ΣDEHTP	0.539 (-1.139;2.218)	-0.07 (-0.715;0.575)	**0.461 (0.014;0.907)****	**0.778 (0.223;1.333)****	0.266 (-0.556;1.087)	'-0.117 (-0.607;0.373)	'-0.187 (-1.137;0.763)	0.726 (-0.351;1.804)
BPA		**0.894 (-0.103;1.89)***	**0.402 (0.029;0.775)****	0.184 (-0.093;0.462)	0.097 (-0.223;0.418)	0.291 (-0.2;0.783)	0.016 (-0.284;0.317)	0.418 (-0.145;0.98)	0.476 (-0.182;1.133)
BPF		1.059 (-0.641;2.76)	0.341 (-0.314;0.996)	0.24 (-0.219;0.7)	0.008 (-0.58;0.597)	0.245 (-0.586;1.077)	0.233 (-0.268;0.733)	0.574 (-0.39;1.537)	0.486 (-0.62;1.591)

The models have been adjusted by maternal age at child birth, number of siblings, child BMI, cotinine level in child urine, child sex, child age at starting school, socio-economic status, place of residence and traumatic events. β-coefficient and 95% Confidence Intervals. ** p<0.05;* p<0.1

Table S10. Joint-pollutant or mixtures model with additional covariate adjustment – Sex-stratified approach

Compound	Metabolite	Sex	Total difficulties	Emotional symptoms	Conduct problems	Prosocial behavior	Hyperactivity/ Inattention problems	Peer relationships problems	Internalizing	Externalizing
			β-coeff (95% CI)							
DMP	MMP	Female	-1.236 (-3.873;1.402)	-0.923 (-2.113;0.268)	-0.015 (-0.687;0.657)	**1.517 (0.799;2.235)****	-0.472 (-1.784;0.839)	0.177 (-0.569;0.924)	-0.742 (-2.343;0.859)	-0.489 (-2.131;1.153)
		Male	0.813 (-2.001;3.627)	0.189 (-0.767;1.146)	0.179 (-0.615;0.974)	**0.885 (-0.11;1.881)***	-0.03 (-1.432;1.372)	0.476 (-0.412;1.363)	0.666 (-0.88;2.211)	0.149 (-1.76;2.057)
DEP	MEP	Female	1.108 (-1.57;3.787)	0.505 (-0.704;1.714)	0.094 (-0.589;0.776)	**-0.63 (-1.359;0.098)***	0.488 (-0.844;1.819)	0.021 (-0.737;0.78)	0.527 (-1.099;2.153)	0.582 (-1.086;2.249)
		Male	1.227 (-2.264;4.718)	0.526 (-0.661;1.713)	0.598 (-0.388;1.583)	-0.244 (-1.478;0.991)	0.732 (-1.007;2.47)	-0.633 (-1.733;0.468)	-0.107 (-2.024;1.81)	1.329 (-1.037;3.696)
BBzP	MBzP	Female	-0.403 (-3.336;2.53)	-0.756 (-2.08;0.568)	**0.739 (-0.009;1.486)***	0.572 (-0.227;1.37)	-0.703 (-2.162;0.755)	0.32 (-0.51;1.15)	-0.433 (-2.213;1.346)	0.034 (-1.792;1.86)
		Male	-3.331 (-7.264;0.602)	**-1.196 (-2.533;0.142)***	-0.929 (-2.04;0.181)	-0.562 (-1.953;0.829)	-1.314 (-3.272;0.644)	0.113 (-1.127;1.353)	-1.081 (-3.242;1.079)	-2.244 (-4.91;0.422)
DiBP	ΣDiBP	Female	**5.921 (1.738;10.103)****	**2.091 (0.208;3.974)****	0.738 (-0.329;1.805)	0.322 (-0.818;1.462)	**2.963 (0.883;5.043)****	0.11 (-1.067;1.287)	**2.195 (-0.333;4.723)***	**3.707 (1.101;6.314)****
		Male	-3.327 (-7.475;0.821)	**-1.24 (-2.65;0.171)***	-0.626 (-1.797;0.545)	-0.587 (-2.054;0.88)	-1.434 (-3.498;0.631)	-0.021 (-1.329;1.286)	-1.26 (-3.539;1.018)	-2.061 (-4.873;0.75)
DnBP	ΣDnBP	Female	**-4.361 (-8.514;-0.208)****	-0.535 (-2.405;1.335)	-0.321 (-1.381;0.738)	**-1.341 (-2.473;-0.208)****	**-2.671 (-4.736;-0.605)****	-0.818 (-1.987;0.352)	-1.351 (-3.862;1.16)	**-2.997 (-5.586;-0.409)****
		Male	0.317 (-5.206;5.839)	0.804 (-1.075;2.683)	0.38 (-1.179;1.939)	0.162 (-1.791;2.115)	0.346 (-2.4;3.091)	-1.219 (-2.959;0.522)	-0.418 (-3.452;2.617)	0.727 (-3.014;4.467)
DEHP	ΣDEHP	Female	-3.821 (-8.649;1.006)	-1.452 (-3.625;0.72)	0.849 (-0.384;2.082)	0.144 (-1.173;1.461)	**-2.5 (-4.902;-0.098)****	-0.69 (-2.047;0.668)	-2.137 (-5.054;0.779)	-1.66 (-4.67;1.349)
		Male	2.409 (-2.311;7.129)	0.164 (-1.441;1.769)	0.065 (-1.267;1.398)	0.299 (-1.37;1.969)	1.438 (-0.911;3.788)	0.735 (-0.752;2.223)	0.9 (-1.692;3.493)	1.505 (-1.694;4.704)
DiNP	ΣDiNP	Female	-0.763 (-4.566;3.04)	0.447 (-1.267;2.162)	**-0.904 (-1.874;0.066)***	**-1.156 (-2.193;-0.119)****	-0.454 (-2.345;1.437)	0.134 (-0.939;1.207)	0.579 (-1.724;2.882)	-1.355 (-3.725;1.014)
		Male	2.536 (-1.687;6.759)	1.253 (-0.183;2.69)*	0.241 (-0.951;1.433)	-0.739 (-2.233;0.754)	0.336 (-1.766;2.437)	0.705 (-0.626;2.037)	1.96 (-0.36;4.281)	0.576 (-2.286;3.438)
DiDP	ΣDiDP	Female	1.191 (-3.164;5.546)	-0.278 (-2.241;1.684)	-0.733 (-1.845;0.378)	0.5 (-0.687;1.687)	**1.995 (-0.172;4.162)***	0.205 (-1.023;1.434)	-0.071 (-2.708;2.565)	1.265 (-1.449;3.979)
		Male	-1.71 (-6.657;3.236)	-1.338 (-3.02;0.345)	0.291 (-1.105;1.688)	0.21 (-1.539;1.96)	0.307 (-2.154;2.769)	-0.972 (-2.531;0.587)	-2.312 (-5.03;0.406)	0.599 (-2.753;3.95)
DINCH	ΣDINCH	Female	-0.491 (-3.939;2.958)	0.338 (-1.218;1.894)	-0.369 (-1.248;0.51)	0.077 (-0.862;1.016)	-0.59 (-2.305;1.125)	0.125 (-0.85;1.1)	0.461 (-1.63;2.552)	-0.959 (-3.107;1.188)
		Male	-1.122 (-4.334;2.089)	0.006 (-1.086;1.098)	-0.368 (-1.275;0.538)	-0.642 (-1.778;0.494)	-1.146 (-2.745;0.454)	0.39 (-0.622;1.402)	0.397 (-1.367;2.161)	-1.515 (-3.692;0.663)

DEHTP	ΣDEHTP	Female	1.448 (-1.416;4.312)	0.14 (-1.151;1.432)	0.273 (-0.457;1.003)	**0.733 (-0.047;1.513)***	0.691 (-0.733;2.115)	0.336 (-0.474;1.145)	0.476 (-1.26;2.212)	0.967 (-0.817;2.75)
		Male	0.507 (-2.962;3.976)	-0.466 (-1.645;0.714)	0.36 (-0.62;1.34)	0.96 (-0.267;2.187)	0.649 (-1.079;2.377)	-0.036 (-1.129;1.058)	-0.501 (-2.406;1.405)	1.009 (-1.343;3.362)
BPA		Female	-0.45 (-3.369;2.469)	-0.353 (-1.67;0.965)	0.107 (-0.637;0.85)	0.374 (-0.421;1.168)	0.105 (-1.346;1.556)	-0.304 (-1.13;0.522)	-0.656 (-2.427;1.115)	0.21 (-1.607;2.027)
		Male	0.33 (-3.118;3.777)	0.464 (-0.708;1.636)	0.362 (-0.612;1.335)	0.901 (-0.319;2.12)	0.206 (-1.512;1.924)	-0.703 (-1.79;0.384)	-0.24 (-2.133;1.653)	0.568 (-1.77;2.906)
BPF		Female	-0.096 (-2.972;2.78)	-0.241 (-1.539;1.057)	0.362 (-0.371;1.095)	-0.315 (-1.098;0.468)	0.19 (-1.24;1.62)	-0.405 (-1.219;0.409)	-0.647 (-2.393;1.098)	0.552 (-1.238;2.342)
		Male	**3.728 (0.762;6.694)****	**1.586 (0.578;2.595)****	0.295 (-0.543;1.132)	0.006 (-1.043;1.055)	0.695 (-0.783;2.174)	**1.15 (0.215;2.085)****	**2.737 (1.108;4.366)****	0.99 (-1.022;3.002)

The models have been adjusted by maternal age at child birth, number of siblings, child BMI, cotinine level in child urine, child age at starting school, socio-economic status, place of residence and traumatic events. β-coefficient and 95% Confidence Intervals. ** $p<0.05$. * $p<0.1$

Table S11. Joint-pollutant or mixtures model with additional covariate adjustment – Sex-adjusted approach

Compound	Metabolite	Total difficulties	Emotional symptoms	Conduct problems	Prosocial behavior	Hyperactivity/Inattention problems	Peer relationships problems	Internalizing	Externalizing
		β-coeff (95% CI)							
DMP	MMP	0.042 (-1.919;2.004)	-0.174 (-0.935;0.588)	0.05 (-0.474;0.574)	**1.292 (0.701;1.884)****	-0.111 (-1.039;0.817)	0.277 (-0.285;0.839)	0.103 (-1.005;1.211)	-0.061 (-1.297;1.175)
DEP	MEP	0.3 (-1.827;2.427)	0.252 (-0.573;1.078)	0.137 (-0.431;0.706)	**-0.603 (-1.245;0.039)***	0.338 (-0.668;1.344)	-0.428 (-1.038;0.181)	-0.176 (-1.377;1.026)	0.475 (-0.865;1.816)
BBzP	MBzP	**-2.004 (-4.354;0.346)***	**-0.91 (-1.821;0.002)***	-0.021 (-0.649;0.607)	0.066 (-0.644;0.775)	**-1.102 (-2.213;0.01)***	0.029 (-0.645;0.703)	-0.881 (-2.208;0.447)	-1.123 (-2.604;0.358)
DiBP	ΣDiBP	0.776 (-2.19;3.741)	0.273 (-0.877;1.423)	0.235 (-0.558;1.028)	-0.096 (-0.991;0.8)	0.466 (-0.937;1.869)	-0.198 (-1.049;0.652)	0.075 (-1.6;1.75)	0.701 (-1.168;2.57)
DnBP	ΣDnBP	-1.462 (-4.752;1.828)	0.019 (-1.257;1.295)	-0.11 (-0.989;0.77)	-0.78 (-1.773;0.214)	-0.778 (-2.334;0.779)	-0.594 (-1.537;0.349)	-0.574 (-2.433;1.284)	-0.888 (-2.961;1.186)
DEHP	ΣDEHP	1.124 (-2.189;4.438)	-0.191 (-1.476;1.095)	0.227 (-0.659;1.113)	0.41 (-0.59;1.411)	0.572 (-0.995;2.14)	0.516 (-0.434;1.466)	0.325 (-1.546;2.196)	0.799 (-1.289;2.887)
DiNP	ΣDiNP	-0.058 (-2.845;2.729)	0.559 (-0.522;1.64)	-0.329 (-1.074;0.416)	**-1.026 (-1.867;-0.185)****	-0.521 (-1.839;0.798)	0.232 (-0.567;1.032)	0.792 (-0.782;2.366)	-0.85 (-2.606;0.906)
DiDP	ΣDiDP	0.959 (-2.205;4.124)	-0.502 (-1.73;0.725)	0.267 (-0.579;1.113)	0.482 (-0.473;1.438)	**1.609 (0.112;3.106)****	-0.414 (-1.321;0.493)	-0.916 (-2.704;0.871)	**1.877 (-0.117;3.871)***
DINCH	ΣDINCH	-1.496 (-3.733;0.741)	0.003 (-0.865;0.871)	-0.482 (-1.08;0.116)	-0.228 (-0.903;0.447)	**-1.107 (-2.165;-0.048)****	0.09 (-0.551;0.732)	0.093 (-1.171;1.356)	**-1.589 (-2.999;-0.18)****
DEHTP	ΣDEHTP	0.036 (-2.129;2.201)	-0.265 (-1.105;0.575)	0.307 (-0.271;0.886)	**0.847 (0.194;1.501)****	-0.009 (-1.034;1.015)	0.003 (-0.618;0.624)	-0.262 (-1.485;0.961)	0.298 (-1.066;1.662)
BPA		0.348 (-1.92;2.616)	0.151 (-0.729;1.031)	0.32 (-0.286;0.926)	**0.59 (-0.094;1.274)***	0.273 (-0.8;1.346)	-0.396 (-1.046;0.254)	-0.245 (-1.526;1.037)	0.593 (-0.836;2.021)
BPF		1.562 (-0.568;3.691)	0.581 (-0.245;1.408)	0.075 (-0.494;0.645)	-0.094 (-0.736;0.549)	0.367 (-0.641;1.374)	**0.538 (-0.073;1.148)***	**1.119 (-0.084;2.322)***	0.442 (-0.899;1.784)

The models have been adjusted by maternal age at child birth, number of siblings, child BMI, cotinine level in child urine, child sex, child age at starting school, socio-economic status, place of residence and traumatic events. β-coefficient and 95% Confidence Intervals. ** p<0.05;* p<0.1

Figure S1. Directed Acyclic Graph of potential confounders of the association between childhood phthalate, DINCH, DEHTP and bisphenols exposure and child behavior (https://dagitty.net/dags.html)

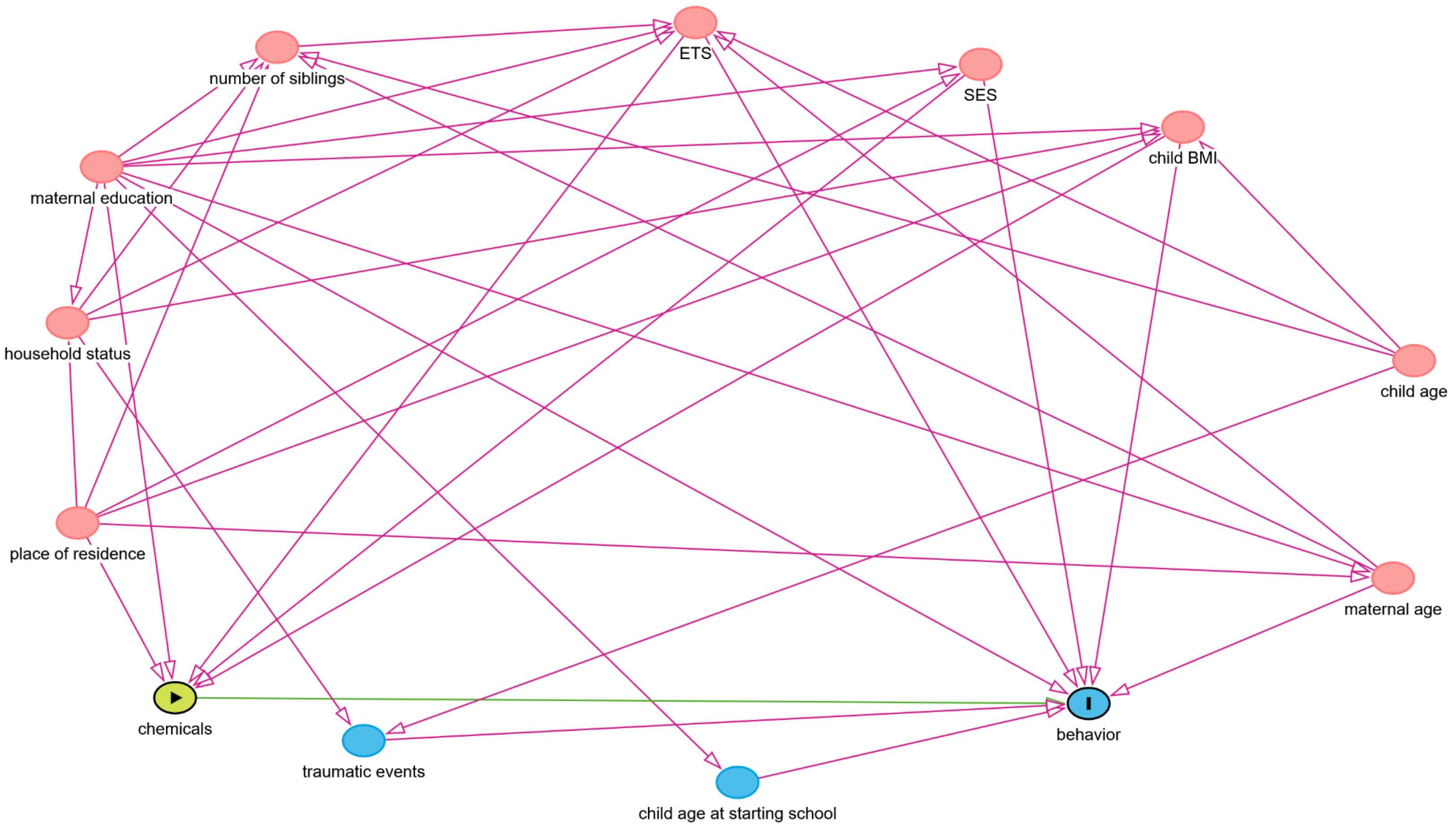

Figure S2. Correlation plot showing the beta-coefficients from multivariable linear *vs.* negative binomial regression models applied on SDQ. Panel A) Single-pollutant exposures model (sex-adjusted and sex-stratified);Panel B) Joint-pollutant exposures model (sex-adjusted and sex-stratified) exposures model.

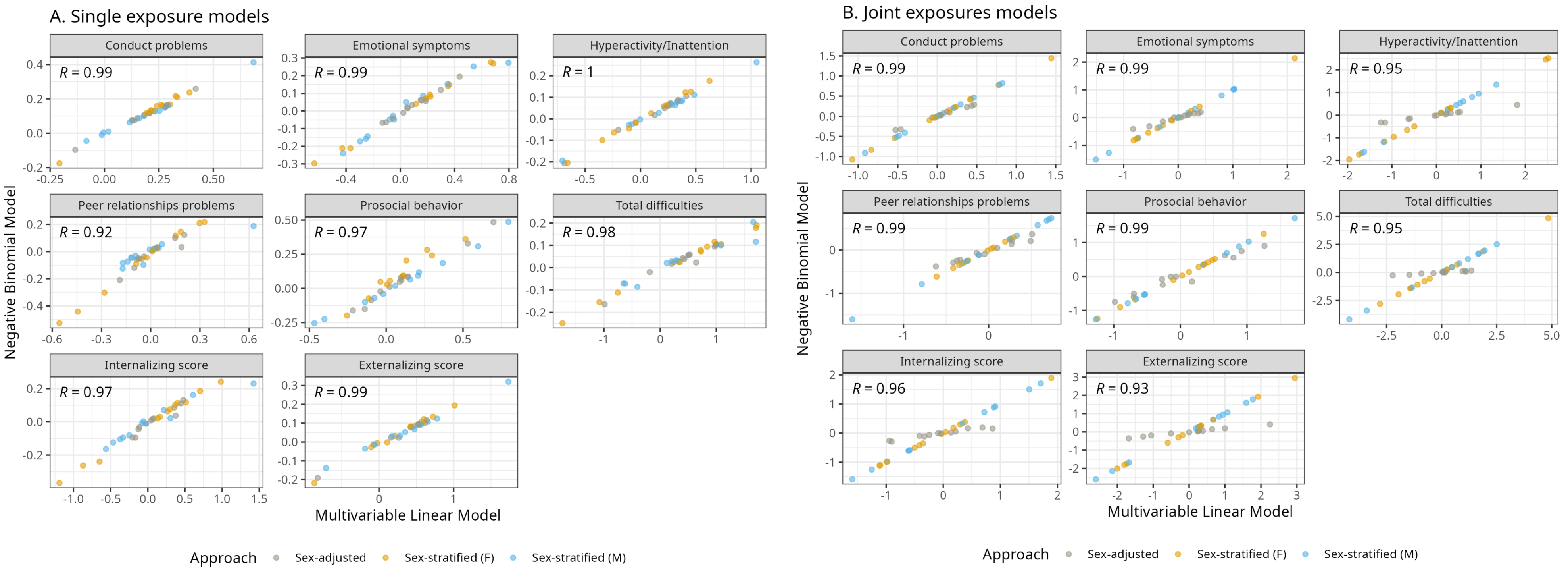

Figure S3. Comparison of Mixtures models from Grouped BayesGWQS package for a single family (left panel) *vs.* BWQS package with single family by default (right panel) applied on SDQ in the sex-stratified approach.

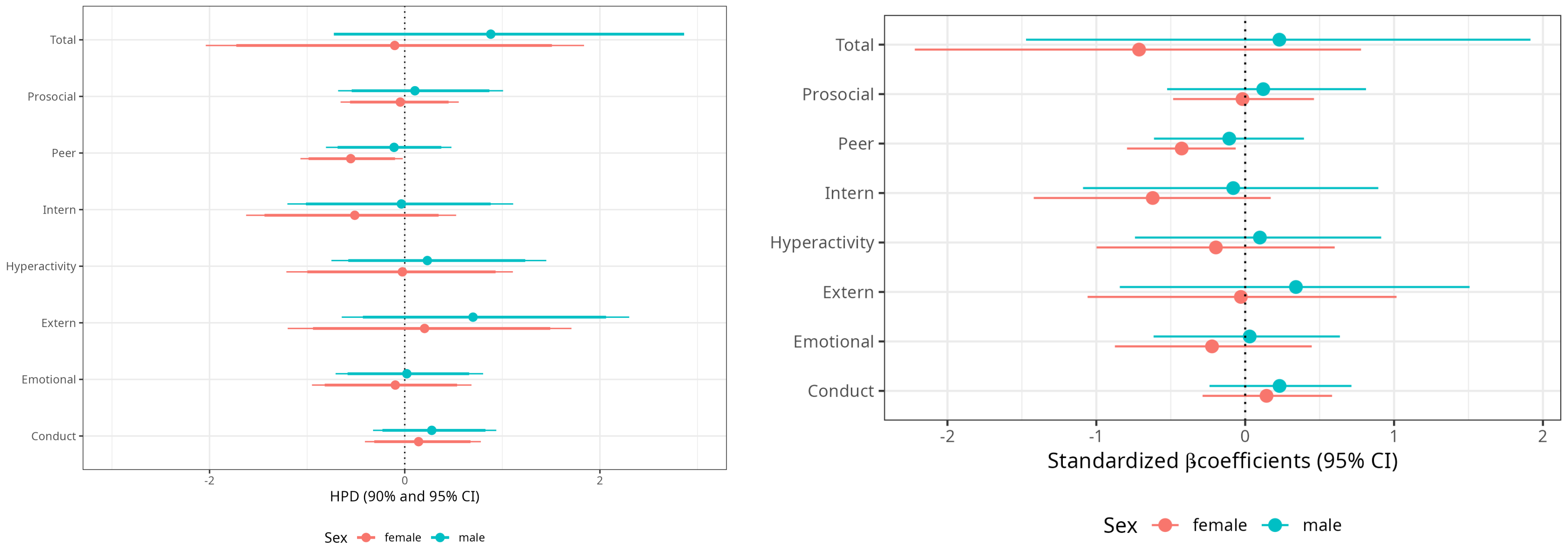

Figure S4. Correlation plot showing the crude cross-sectional associations between SDQ scales and concentrations of phthalates, DINCH, DEHTP and bisphenols (BPA and BPF), by sex

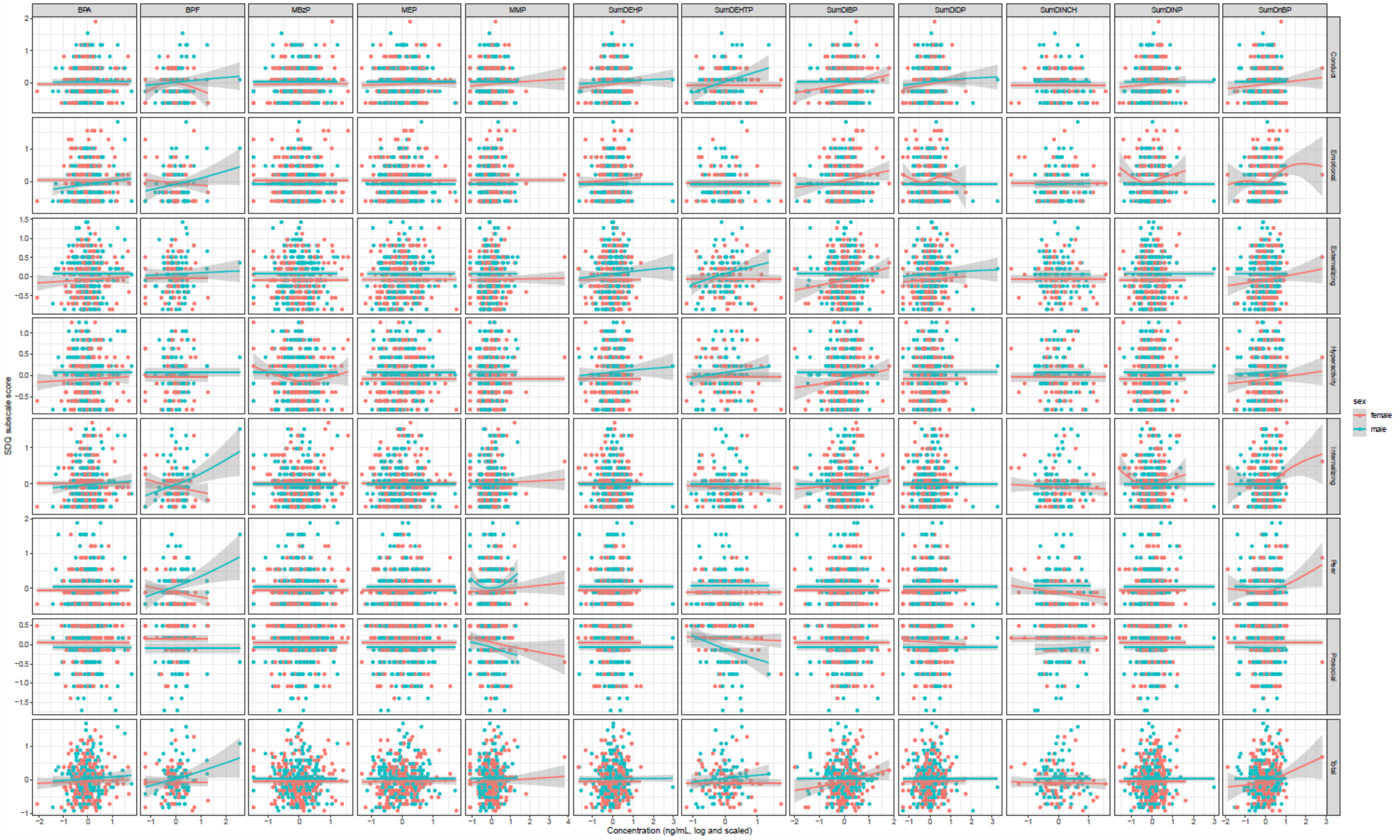

Supplement: Supplementary file 1 — Supplementary Material 1: Appendix A. Supplementary data. Supplementary data to this article can be found online [file 12940_2025_1210_MOESM1_ESM.pdf]
